# Public contestation over agricultural pollution: a discourse network analysis on narrative strategies in the policy process

**DOI:** 10.1007/s11077-021-09439-x

**Published:** 2021-10-19

**Authors:** Simon Schaub

**Affiliations:** Institute of Political Science, Heidelberg University, Bergheimer Straße 58, 69115 Heidelberg, Germany

**Keywords:** Narrative policy framework, Discourse network analysis, Public policy, Agriculture, Water pollution, Nitrate

## Abstract

The overuse of fertilizers in agriculture and their entry into freshwater has many negative impacts on biodiversity and poses problems for drinking water resources in Germany. In response to exceeding levels of nitrate concentrations in groundwater in parts of the country, an intense public dispute evolved and a significant policy change in fertilizer regulation occurred in 2020. Based on the German case of agricultural water pollution, this study demonstrates in an innovative way how discourse network analysis is a fruitful method for the integrated study of actor coalitions and their use of narrative strategies in public debate. Theoretically, the study draws on the narrative policy framework (NPF) to explain how actor coalitions use narrative strategies to attempt to influence policymaking on water pollution by agricultural activities. The empirical analysis builds on newspaper articles and press releases disseminated between 2010 and 2020. The results demonstrate how two opposing actor coalitions with congruent policy beliefs formed in the struggle over fertilizer regulation. These not only diverged in their policy beliefs but also differed in their use of narrative strategies to try to expand or contain the policy issue. More precisely, the coalitions adapted their narratives over time in response to changes in the likelihood to win or lose. Furthermore, the results suggest the coalition in favor of stricter fertilizer regulation was more sophisticated in its effort to mobilize specific target groups. Overall, the article provides a valuable contribution to the literature on the NPF by combining research on coalition formation and policy narratives.

**Supplementary Information:**

The online version contains supplementary material available at 10.1007/s11077-021-09439-x.

## Introduction

Intensification of agriculture is increasingly causing problems for water protection (Baker et al., 2017; van Grinsven et al., 2006). Although nitrogen is an important component in agriculture to enhance production, overuse of manure and artificial fertilizers has many negative impacts on social-ecological systems (Erisman et al., 2013; Kirschke et al., 2019). Fertilizer overuse threatens the health of water resources. Runoff into surface waters causes eutrophication (excessive growth of plants and algae) which harms biodiversity (Baker et al., 2017), while increased nitrate levels in groundwater pose growing problems to drinking water production (van Grinsven et al., 2006).

The conflict between water protection and agricultural interests has led to intense public dispute over fertilizer regulation in Germany in the last years. In fact, Germany has recently experienced one of the strongest waves of farmer protests since decades. Especially small-peasant farmers mobilized and protested against higher environmental standards, including stricter fertilizer regulation (Agrarheute, 2019; Spiegel Online, 2019). This group of farmers has come under significant economic pressure, documented by a constant decrease in the number of small family farms and increased consolidation of the German agricultural sector (BMEL, 2019). On the other side, a coalition of water associations and environmental organizations started a joined public campaign to raise awareness on freshwater pollution from agricultural nitrates and mobilize for stricter fertilizer regulation (BDEW, 2018; BUND, 2019). The surrounding public debate changed over the course of a decade from rather technical to political, characterized by emotion and blame.

The *narrative policy framework (NPF)* provides a useful framework to investigate how political actors use public debate as an arena to influence policymaking (Jones, 2018; Jones & McBeth, 2010; McBeth et al., 2010; Shanahan et al., 2013; Stauffer & Kuenzler, 2021; Tosun & Schaub, 2021; Vogeler et al., 2021b). The framework suggests that political actors structure their policy narratives strategically to influence policy outcomes (Shanahan et al., 2011). The NPF is therefore well suited to analyze the public dispute over agricultural nitrate pollution of freshwater in Germany and investigate whether and how supporters and opponents of stricter fertilizer regulation participated strategically in the public debate to influence the policy outcome in their interest.

To this end, the article builds on previous research on the NPF, public mobilization and coalition formation, and poses the following two research questions: Are there differences between actor coalitions in their participation in the public debate and use of narrative strategies? Do coalitions adapt their behavior over time in response to changes in likelihood of winning or losing on the policy issue?

Several objectives are pursued in this article. Theoretically, it incorporates arguments on strategic participation in public debates into the study of policy narratives and narrative strategies. There is reason to expect that actor coalitions differ in how actively they participate in the public debate (Schaub & Metz, 2020; Tosun & Schaub, 2017), which may have an impact on the observed policy narratives. Based on earlier work by Schattschneider (1960) and Baumgartner and Jones (1993), I argue that coalitions differ in their incentive to participate in the public debate depending on whether they seek to defend a policy monopoly or mobilize for policy change, which should be especially relevant in cases of low issue salience at the onset of political conflicts. This leads to hypothesize that actors mobilizing for policy change participate more heavily in public debate, whereas those defending the policy monopoly are expected to refrain from participation. Furthermore, the article mostly investigates changes in behavior over time. To this end, it expects increases in the likelihood to lose a policy conflict to result in increasing participation whereas it expects increases in the likelihood to win to lead to lower participation. Similarly, I build on earlier work on the NPF and hypothesize coalitions to use increasingly narrative strategies intended to expand the policy issue when the likelihood to lose increases and with increasing likelihood to win to use increasingly narrative strategies aimed at containing an issue. Conceptually, this study adds clarity to the use of the NPF at the meso-level (i.e., the behavior of groups) by more clearly distinguishing between the analysis of coalition formation and coalitions’ use of narrative strategies. I argue that the formation of coalitions should be measured separately from narrative strategy, e.g., based on congruent belief systems (Weible et al., 2020). Previous studies often measured coalition formation based on actors’ usage of narrative policy elements, such as characters, policy symbols or frames. However, actors with congruent policy beliefs do not necessarily engage in concerted action to achieve their policy goals (Nohrstedt & Olofsson, 2016; Weible et al., 2020).

To achieve the former, the article makes a major methodological contribution. Building on earlier suggestions (Leifeld, 2017; Shanahan et al., 2013; Weible et al., 2016), this study shows that *discourse network analysis* is a useful method to analyze coalition formation within a policy subsystem, identify members of clearly separable coalitions and investigate whether coalitions differ in their level of activity in the public debate and their use of narrative strategies. Discourse network analysis has been developed explicitly to study coalition formation based on congruent policy beliefs (Leifeld, 2016) and has been applied in the study of various policy subsystems (Leifeld, 2013, 2020; Rinscheid, 2020; Tobin et al., 2018; Tosun & Schaub, 2017). Investigating discourse networks and the relationships between actors and narrative elements enables an enhanced analysis of the relational dimension inherent in the NPF.

Finally, the study makes an empirical contribution on the nexus of water and agriculture in Germany. It contributes to better understanding existing conflict lines in the German policy subsystem of agricultural nitrate water pollution, provides a systematic analysis of the different positions taken in the public debate, and provides insight on how supporters and opponents of stricter fertilizer regulation try to influence policymaking in Germany.

The article proceeds by introducing the empirical case of agricultural nitrate pollution in Germany. It subsequently presents the theoretical argument on participation in the public debate, coalition formation and narrative strategies and derives theoretical hypotheses. Then, after explaining the methodology used to collect and analyze the empirical data, the article presents and discusses the empirical findings. The final section provides concluding remarks and points for further research.

## Empirical case: agricultural nitrate pollution of water in Germany

Agricultural nitrate pollution has increasingly become a salient environmental and agricultural policy issue in Germany over the last decade. While media reporting on the issue was low in 2010, it has sharply increased over time.^1^ The public debate reflected by media coverage has been characterized by a growing adversarial dispute between different stakeholders: environmental groups, water associations and the Green Party mobilizing for stricter regulation, and mainly farmer associations opposing stricter regulation and advocating liberalization of legal provisions.

Nitrate runoff in Germany mostly results from agricultural activities (Knoll et al., 2020; Kunkel et al., 2017), is especially high in regions with high livestock density (Kastens & Newig, 2007) and places significant pressure on German ground- and surface waters (European Environment Agency, 2018). To counter nitrate water pollution in all European member states, the EU passed several directives: the *Water Framework Directive (WFD; *Directive, 2000*/60/EC),* the *Nitrates Directive (ND; Directive 91/676/EEC)* and the *Groundwater Directive (GWD; Directive 2006/118/EC)* define nitrate concentration limits for groundwater bodies and request EU member states to reduce the level of agricultural nitrate pollution in case the limits are exceeded. The *German Surface Water Ordinance (OGewV)*, adopted in 2011, and the *Federal Water Act (WHG)*, adopted in 2009, represent the main water legislation at the federal level and transpose the WFD into domestic law (Berger, 2017). The *Fertilizer Act (DüG)* and the *Fertilizer Ordinance (DüV)* transpose the ND into German law. The DüG regulates the manufacture, placing on the market and application of fertilizers and the DüV sets out the usage criteria in accordance with good agricultural practice and, thus, defines how the DüG is put into practice (Umweltbundesamt, 2019b). The DüV has mostly been subject of the debate on stricter fertilizer regulation to reduce nitrate pollution and meet the required nitrate concentration levels since it defines in detail how reduction is to be achieved.

Although Germany has adopted legislation on fertilizer use and water protection, nitrate concentration levels in Germany have exceeded legal concentration limits (European Environment Agency, 2018). Consequently, the *European Commission (EC)* has repeatedly accused Germany of not sufficiently addressing agricultural nitrate pollution and failing to adequately transpose the EU directives on nitrate pollution into domestic law (European Commission, 2019).

Changes to the German fertilizer legislation occurred only gradually after increasing pressure by the EC. In fact, policymaking in Germany on the issue of agricultural nitrate pollution exemplifies well what Zohlnhöfer and Tosun (2021) describe as the new German policy style of *exclusive incrementalism*, where existing policies are mostly changed incrementally and in response to exogenous events or public opinion. In July 2014, the EC opened a first infringement procedure against Germany for failing to meet obligations under the ND. After Germany did not respond adequately, the EC referred the case to the *Court of Justice of the European Union (CJEU)* in April 2016 (European Commission, 2019). In response, Germany revised the DüV in June 2017, but did not significantly change the criteria on fertilizer use in agriculture (Härtel, 2018; Taube, 2018). In the meantime, a first ruling by the CJEU in June 2018 found implemented measures in the DüV prior to its legal revision insufficient.^2^ The CJEU ruling and the EC evaluation of the DüV as insufficient led the EC to threaten Germany with a second infringement procedure. The EC sent a second letter of formal notice in July 2019 urging Germany to implement adequate regulation until May 2020. This represents a turning point in recent policymaking on nitrate pollution in Germany. The threat to initiate a second infringement proceeding (potentially resulting in fines of up to 850,000 € per day) led to an immediate response by the federal ministry of agriculture (BMU) and the federal ministry of the environment (BMEL) which promised to propose a significant revision to the DüV. The ministries are typically divided on conflicts between agricultural and environmental policy goals (Tosun et al., 2019) and have been divided on the issue of nitrate pollution. For the first time, both ministries started to publish joined press releases after July 2019 on nitrate pollution, with later agreement on a revision proposal for the DüV in September 2019. Only after the EC finally signaled its satisfaction with the proposal, both German chambers of parliament adopted a revised DüV in March 2020.

The increase in issue salience and the temporal change in external conditions make the empirical case very well suited for investigating coalition formation in the policy subsystem and the adaptation of narrative strategies to influence policy outcomes. This study therefore focuses on the German public debate on agricultural nitrate water pollution between January 2010 and December 2020.

## Theoretical approach

In general, the public debate represents one venue political actors use to try to influence policymaking (Leifeld, 2013, 2020). Research has identified several ways in which participation in public debates may be influential: First, research on agenda-setting shows that emphasizing certain problem perceptions or policy solutions increases a topic’s likelihood to be discussed in legislative institutions (Baumgartner & Jones, 2009; Soroka & Wlezien, 2009; Tosun & Scherer, 2020; Tosun & Varone, 2020). Second, disseminating new information in public debates can lead to policy learning across political actors, which may trigger policy change (Leifeld & Brandenberger, 2019; Sabatier, 1998). Third, influencing public debates can affect public opinion, which has been found to affect the behavior of decision makers (Burstein, 2003; Mühlböck & Tosun, 2018; Shapiro, 2011; Soroka & Wlezien, 2009). To achieve these policy goals, political actors use social media to directly participate in the public debate (Bossner & Nagel, 2020; Gupta et al., 2016; Lybecker et al., 2015; Merry, 2016, 2018; Parth & Nyby, 2020) or they publish press releases with content intended to be reproduced and disseminated by traditional mass media, such as newspapers (Leifeld, 2013; Merry, 2019; Schaub & Braunbeck, 2020). Political actors’ use of media is well reasoned, as it evidently affects public opinion and agenda-setting (McCombs & Valenzuela, 2021).

Whether and how political actors participate depends on strategic considerations (Schaub & Braunbeck, 2020; Schaub & Metz, 2020; Shanahan et al., 2011; Tosun & Schaub, 2017). The NPF is a useful framework for understanding *how* political actors participate in public debates. The framework builds on the idea that political actors construct their policy narratives strategically to influence policymaking. Moreover, it has been developed by a group of scholars to investigate the role of policy narratives at different levels, from micro- to macro-level (Boscarino, 2019; Jones, 2018; Jones & McBeth, 2010; McBeth et al., 2007; Shanahan et al., 2011, 2018). Whereas studies on the micro-level focus on individuals, those on the macro-level investigate cultural and institutional phenomena. At the meso-level, research on the NPF investigates the behavior of actor coalitions. The framework strongly builds on the *advocacy coalition framework (ACF)* in its conceptualization of coalitions (Shanahan et al., 2011). It adopts the idea that political actors form actor coalitions within policy subsystems based on congruent policy beliefs and their preferred policy outcome (Sabatier & Jenkins-Smith, 1993; Shanahan et al., 2011).

The NPF assumes that political narratives, created by a variety of political actors, play an important role in policy processes and transmit political actors’ policy beliefs and policy goals into policy outputs (Shanahan et al., 2011). Conceptualized from a structuralist perspective, policy narratives are characterized by common elements and can be generalized across different situations. These elements also define a policy narrative and make it distinguishable from other non-political communication, e.g., technical reports. A policy narrative typically is situated within a specific setting or policy context, has a plot, contains several characters, includes a policy stance (such as an endorsement for or rejection of a policy solution), policy beliefs and is disseminated by political actors to attain their preferred policy outcome (Jones & McBeth, 2010). It may further contain additional narrative elements, such as policy symbols, policy surrogates, different causal mechanisms, evidence, or cost–benefit frames (Jones, 2018; Jones & McBeth, 2010; Shanahan et al., 2013). A policy narrative’s plot ties these various narrative elements together. For instance, it connects different characters: *villains* are accused of harming *victims* and *heroes* are portrayed to solve problems for certain *beneficiaries* (Jones & McBeth, 2010; Weible et al., 2016).

A central idea of the NPF is that political actors use these narrative elements as a form of *narrative strategy* to expand or contain the *scope of conflict*, a notion derived from classical work by Schattschneider (1960). Political actors with an interest in policy change are expected to try to expand the political conflict by increasing attention on a policy issue. Thereby, they hope to gain new political allies and public support. In contrast, actors defending the policy status quo will try to contain the political conflict and diminish attention. This is especially relevant for cases Schattschneider considered as *normal politics*, where initially issue salience is low and a small group of political actors, who control the definition of the policy problem and the policy outcome, try to defend their *policy monopoly* (Baumgartner & Jones, 1993; Schattschneider, 1960; Stephan, 2020). Similarly, the NPF assumes policy narratives to contain mainly two broad types of plots based on work by Stone (2002): a *plot of decline*, which spins a tale of a deteriorating situation to expand a policy conflict, and a *plot of control,* constructed to contain a policy conflict. The latter is intended to convey the message that a situation is under control and does not need further attention or any other policy solutions. Constructing a plot of decline is associated with actors perceiving themselves as losing on a policy issue whereas the plot of control is expected to coincide with the perception of winning (Shanahan et al., 2013).

### Coalition formation and participation in the public debate

Before turning to *how* coalitions differ in their use of narrative strategies, I argue that it is important to first investigate *whether* political actors participate in the public debate or avoid participation for strategic reasons. In fact, I contend that we can expect coalitions to differ in how strongly they resort to the public debate as a venue to influence policymaking. Also based on Schattschneider’s *scope of conflict* and further work by Baumgartner and Jones (1993), Tosun and Schaub (2017) and Schaub and Metz (2020) suggest that coalitions with an interest in defending a policy monopoly and preserving the policy status quo *initially* avoid participation in the public debate to keep the level of public attention low. Instead, they may incline to other, less noisy venues, e.g., lobbying, to influence policymaking. In contrast, coalitions with an interest in policy change should participate more actively in the public debate to create attention on a policy issue and break the policy monopoly. In addition, policy actors within the latter coalition often possess only limited access to decision makers or find themselves in a weak bargaining position, which makes public debates a comparatively easy venue to access (Johannesson & Weinryb, 2021; Schaub & Metz, 2020). Thus, we should observe differences between coalitions in how actively they participate in the public debate at the onset of political conflicts when issue salience is still low.

Regarding the empirical case at hand, it is reasonable to expect the formation of two main adversarial coalitions over time: one mobilizing for and the other against stricter fertilizer regulation. Since legislation on the issue was comparatively liberal in 2010, the *status quo coalition* against stricter regulation should be less inclined to participate in the public debate in order to diminish attention and preserve the policy status quo at this time. In contrast, the *reform coalition* in favor of stricter regulation should have a stronger incentive to resort to the public debate to increase attention on the issue. These considerations lead to the first hypothesis:

#### H1a

The coalition mobilizing for stricter regulation will participate more strongly in the public debate than the coalition defending the policy status quo at the onset of a political conflict.

Initially, the *status quo coalition* should perceive itself as more likely to defend the policy status quo and win on the policy issue, since policy monopolies tend to be stable over time (Baumgartner & Jones, 1993). Agricultural policy in Germany has predominantly served the interests of farmers in recent decades and has strongly been determined by *exceptional* institutional arrangements (Daugbjerg & Feindt, 2017; Feindt, 2018; Tosun, 2017). Therefore, the chance of the *reform coalition* breaking the policy monopoly is low and this coalition should perceive itself as more likely to lose on the policy issue. Contrary to the status quo coalition, the reform coalition has nothing to lose and can only win when participating in the public debate.

Following this reasoning, a change in a coalition’s perception of whether it is losing or winning on a policy issue also changes its rationale to participate strongly in a public debate or reduce their participation. The more the status quo coalition fears it is losing on a policy issue, the more strongly it should be inclined to change its behavior toward expanding the scope of conflict and participating more frequently in the public debate. In contrast, the more the reform coalition perceives itself to be winning on the issue, the more it will be inclined to contain the scope of conflict.

Regarding the empirical case, the increasing pressure exerted by the EC to adopt stricter fertilizer regulation over time gradually increases the likelihood for significant policy change and, thus, the status quo coalition’s chance to lose and the reform coalition’s chance to win on the policy issue. Consequently, the gradual change in the external conditions should decrease the status quo coalition’s perceived likelihood to win on the policy issue. In contrast, the changing conditions should increase the reform coalition’s likelihood for winning. Consequently, the status quo coalition will be more and more inclined to participate in the public debate whereas the reform coalition will tend to reduce its activity. These considerations lead to the next two hypotheses:

#### H1b

An increase in a coalition’s perceived likelihood to lose on a policy issue will lead to more frequent participation in the public debate.

#### H1c

An increase in a coalition’s perceived likelihood to win on a policy issue will lead to less frequent participation in the public debate.

### Narrative strategies

The NPF suggests that coalitions differ not only in their level of participation in the public debate, but also in *how* they participate in the debate. More specifically, the NPF suggests that coalitions construct their policy narratives in a way to either expand or contain the policy conflict, and that coalitions differ in these narrative strategies depending on whether they perceive themselves as winning or losing on a policy issue (Gupta et al., 2014; Merry, 2019; Shanahan et al., 2013).

This study focuses on two types of narrative strategies, which have repeatedly been found to be used by coalitions to shape the scope of conflict: first, cost–benefit frames and, second, the use of characters (Gupta et al., 2014; McBeth et al., 2007; Merry, 2019; Shanahan et al., 2013).

#### Cost–benefit frames

Political actors frame issues in a strategic way to convince others of their views and interpretations. The use of frames is based on the idea that these actors do not simply communicate political or social realities, but construct realities by selecting and highlighting certain aspects of an issue, such as a particular problem perception or causal interpretation, in a way to persuade others of their views (Entman, 1993; Matthes, 2012). The use of frames in communication evidently impacts issue salience and public opinion (Druckman et al., 2012; Vreese et al., 2011).

Research on policy narratives shows political actors use cost–benefit frames to shape the scope of conflict (McBeth et al., 2007; Shanahan et al., 2011; Stephan, 2020). Coalitions perceiving themselves as losing on a policy issue tend to *diffuse the costs* and *concentrate the benefits* of an opposed policy solution to expand political conflict. When *diffusing costs*, coalitions overemphasize the costs inflicted upon the broader public interest to involve more stakeholders in the policy conflict and gain their support (McBeth et al., 2007; Shanahan et al., 2011; Stone, 2002). In the present case, the reform coalition may portray nitrate pollution of water bodies as not only a problem for water providers or ecosystems, but also for citizens (taxpayers) or future generations due to potential contamination of drinking water resources. In contrast, the status quo coalition may accentuate costs resulting from stricter fertilizer regulation by overemphasizing consequences for food security in Germany. When *concentrating benefits*, any potential benefits of the opposed policy solution are downplayed and attributed to narrow special interests to demobilize the opposition (McBeth et al., 2007; Shanahan et al., 2011; Stone, 2002). This specific combination of frames is typically part of a *plot of decline* and termed the *loser’s tale* as it is usually associated with coalitions who perceive themselves as losing on a policy issue (McBeth et al., 2007). In contrast, the *winner’s tale* involves strategies to contain political conflict. Coalitions perceiving themselves as winning tend to *diffuse the benefits* of their advocated policy outcome and *concentrate the costs*. When *diffusing benefits*, coalitions portray their preferred policy solution as one that serves the public interest and they downplay any disadvantages by *concentrating the costs*. The winner’s tale is typically part of a *plot of control* to diminish attention on a policy issue (Jones & McBeth, 2010; McBeth et al., 2007; Shanahan et al., 2011).

Table 1 gives an overview of the eight different frames coalitions may use to either expand or contain the scope of conflict, depending on whether they perceive themselves as losing or winning on the policy issue and whether they mobilize for stricter regulation or advocate liberalization.

**Table 1:** Cost–benefit frames as narrative strategies

	Reform coalition	Status quo coalition
Expansion (loser’s tale)	Diffuse costs of liberalization	Diffuse costs of stricter regulation
	Concentrate benefits of liberalization	Concentrate benefits of stricter regulation
Containment (winner’s tale)	Diffuse benefits of stricter regulation	Diffuse benefits of liberalization
	Concentrate costs of stricter regulation	Concentrate costs of liberalization

Based on Shanahan et al. (2011) and Shanahan et al. (2013)

The above reasoning leads to the following hypothesis:

##### H2a

Coalitions which are likely to lose on a policy issue will predominantly use expanding cost-benefit frames and those who are likely to win on a policy issue will predominantly use containing cost-benefit frames.

Similar to changing their participation in the public debate in response to a variation in the perceived likelihood to win or lose on a policy issue, coalitions are expected to adapt their narrative strategies as well. An increase in the perceived likelihood to lose on the policy issue should urge coalitions to attract attention on the issue by increasing the use of expanding frames and reducing containing frames. In contrast, an increase in the perceived likelihood to win on the policy issue should lead coalitions to reduce their efforts to attract attention on the issue, by reducing the use of expanding frames and increasing containing frames. This leads to two complementary hypotheses:

##### H2b

An increase in a coalition’s perceived likelihood to lose on a policy issue will lead the coalition to use expanding frames more frequently and containing frames less frequently in their policy narratives.

##### H2c

An increase in a coalition’s perceived likelihood to win on a policy issue will lead the coalition to use containing frames more frequently and expanding frames less frequently in their policy narratives.

#### Characters

The second type of narrative strategy used to shape the scope of conflict relates to how coalitions populate their policy narratives with characters. Here, two strategies are distinguished: on the one hand, coalitions might make use of a *devil shift* when they feel threatened and likely to lose on the policy issue (Schlaufer, 2018; Shanahan et al., 2013). The devil shift is a notion borrowed from the ACF, which describes a situation where opposing coalitions overemphasize the power and the *evilness* of their opponents (Sabatier et al., 1987; Shanahan et al., 2013). Thus, the NPF suggests that policy narratives characterized by a devil shift predominantly emphasize the role of villains who cause problems and inflict damage upon victims, connected through a plot of decline (Shanahan et al., 2013). Casting the opposition as villains aims at demobilizing the opposition while emphasizing the harm caused to victims is intended to generally increase attention on the issue and to gain victims’ support (Shanahan et al., 2013). Since the devil shift is used to expand the political conflict, coalitions which perceive themselves as losing are more likely to use this strategy.

In contrast, winning coalitions are associated with an *angel shift*, where they predominantly portray themselves or their allies as heroes who are able to fix the problem to the benefit of certain beneficiaries (Shanahan et al., 2013; Weible et al., 2016). Emphasizing the role of heroes while avoiding the use of villains is part of a plot of control aimed at containing a policy issue. It is used to convey the message that everything is under control and the coalition is able to solve the policy issue (Shanahan et al., 2013). The above reasoning leads to the following hypothesis:

##### H3a

Coalitions which are likely to lose on a policy issue will use the devil shift and coalitions which are likely to win on a policy issue will use the angel shift.

Analogously to hypotheses 2b and 2c, coalitions are expected to change their use of characters in response to changes to their perceived likelihood to win or lose on the policy issue. An increase in the perceived likelihood to win on the policy issue should allure coalitions to use heroes and beneficiaries more frequently and an increase in the perceived likelihood to lose to emphasize harm caused by villains to victims. This leads to an additional pair of complementary hypotheses:

##### H3b

An increase in a coalition’s perceived likelihood to lose on a policy issue will lead the coalition to use villains and victims more frequently and heroes and beneficiaries less frequently in their policy narratives.

##### H3c

An increase in a coalition’s perceived likelihood to win on a policy issue will lead the coalition to use heroes and beneficiaries more frequently and villains and victims less frequently in their policy narratives.

## Data and methods

This study uses a longitudinal case study to test the hypotheses on coalition formation and coalitions’ use of narrative strategies derived in the previous section. The observation period ranges from January 2010 until December 2020. The starting point was chosen based on the criteria of issue salience and media coverage. While collecting the data, it proved that both issue salience and media coverage were low before 2010 (see Fig. 4 for media coverage within the observation period). The ending point represents the most recent data. The observation period is subdivided into four separate periods for analytical reasons. Three different criteria with successive levels of priority guided the identification of these periods: First and most importantly, changes in the external environment, which are expected to change coalitions’ perceptions of their chances to win or lose on the policy issue, mark the start and/or end of each period. Second, every period needs sufficient observations to measure coalition formation and test differences between coalitions’ narrative strategies robustly. Third, the period lengths should be about equal. This led to four different periods: a first period from the beginning of January 2010 until the end of June 2014, which marks the start of the first infringement proceeding against Germany. The second period starts in July 2014 and ends in March 2017 when the first revision of the fertilizer ordinance was adopted. The third period starts in April 2017 and ends on July 24, 2019, when the EC threatened to open a second infringement procedure. The fourth period subsequently begins on July 25, 2019, and covers the debate until the end of December 2020.

The German case on agricultural nitrate pollution of water bodies represents a very good case for investigating and illustrating the expected differences between coalitions and changes in their behavior over time for several reasons. First, investigating the association between winning or losing on a policy issue and narrative strategies has been difficult, because it requires the measurement of a coalitions’ perceived likelihood to win or lose, which is complicated (Gottlieb et al., 2018). The great advantage of the case at hand is the gradual change in the external environment: the increasing pressure by the EC to tighten the fertilizer regulation should have led to respective changes in both coalitions’ perceived likelihood to win or lose over time. As portrayed in section two on the empirical case, there is large support for this assumption. Therefore, it is possible to investigate whether coalitions responded to this change in likelihood by adapting their narrative strategies over time. Consequently, it is not necessary to *absolutely* determine which coalition was winning or losing at a certain point in time. If coalitions consistently adapted their behavior at points in time *relative* to previous points in time (more expansion in response to higher likelihood to lose; more containment in response to higher likelihood to win), then this would indicate a possible causal association between the likelihood to win or lose and narrative strategies (hypotheses 2b, 2c, 3b and 3c). The same applies for the expected association between the likelihood to win or lose and changes in participation in the public debate (hypotheses 1b and 1c). Second, the course of the public debate is well suited to investigate coalition formation. The debate became increasingly politicized over time. Therefore, it is well suited to observe the participation of political actors in the public debate and identify adversarial coalitions. Third, the observation period captures the begin of the public dispute and, therefore, allows the investigation of the expected differences between coalitions at the onset of the political conflict (hypothesis 1a).

### Data collection

The data for the analysis were collected in two steps. First, newspaper articles published in the *Frankfurter Allgemeine Zeitung (FAZ)* served to determine political actors in the public debate on nitrate pollution in Germany between January 2010 and December 2020. The *FAZ* represents one of the principal nationwide newspapers in Germany, corresponds well with the “quality press” criterion of wide circulation, reputation and moderate political positioning (Barranco & Wisler, 1999), and has proven to be a reliable data source for discourse network analysis in Germany (Leifeld, 2013; Schaub & Braunbeck, 2020; Tosun & Lang, 2016). A keyword search was used to select only newspaper articles dealing with the issue of nitrate pollution of water bodies in Germany.^3^ The final sample consists of 190 newspaper articles. Within these articles, 31 political actors were identified based on the following definition: political actors are organizations from inside or outside of government who participate in the formulation and implementation of public policy or regularly try to influence the policy output and policy outcome in their interest (Janning et al., 2009; Weible et al., 2020). Since the study is interested in actors’ behavior in the public debate over time, the final sample included only those political actors who regularly participated in the public debate. As a prerequisite for this, actors needed to make a public statement on nitrate pollution at least at two different points in time during the observation period [see also Leifeld (2017) on selecting actors in the study of public debates].

The newspaper articles were complemented by press releases published by the 31 political actors. These are better suited to capture political actors’ narrative strategies since they contain original, unabridged text written by the actors themselves. In total, 554 press releases were added to the 190 newspaper articles resulting in a final sample of 744 documents.

### Data analysis

Methodologically, the study proceeded in two main steps. First, *discourse network analysis* (Leifeld, 2016, 2017) was applied to analyze coalition formation within the policy subsystem and whether identified coalitions differed in their participation in the public debate (hypotheses 1a – 1c). Second, the study used a mix of quantitative and qualitative analysis to investigate how the actor coalitions determined in the first step constructed their policy narratives (hypotheses 2a–3c).

### First part of the empirical analysis: coalition formation over time

The first part of this study used discourse network analysis to study coalition formation and determine political actors’ membership in coalitions based on their policy beliefs. The method combines qualitative content analysis with social network analysis and was explicitly developed to identify actor coalitions within policy subsystems based on political actors’ congruent policy beliefs (Leifeld, 2016). Actors’ policy beliefs were measured via *statements* they articulated in the public debate at different points in time. Therefore, discourse network analysis not only allows determining the number of coalitions and their cohesiveness, but also how these coalitions change over time. *Statements* are text portions where actors indicate support for or opposition to different *concepts* (Leifeld, 2013). In this case, actors’ positions toward *concepts* represented a measure of their policy beliefs.

This study identified coalitions based on two of the three types of policy beliefs originally put forth by the ACF: actors’ *policy core beliefs* and *secondary* (or *instrumental*) *beliefs* (Sabatier, 1998). Policy core beliefs tend to be stable over time, are related to a specific policy subsystem and can be normative and empirical in nature. Typical examples of policy core beliefs are problem perceptions, causal understandings and policy positions. Secondary beliefs are at a more specific level and refer to the means of achieving policy goals, such as specific policy instruments. These beliefs are more likely to change over time (Sabatier, 1998; Weible & Jenkins-Smith, 2016).

Table 2 lists the different policy beliefs coded in this study. These were captured through binary variables indicating agreement or disagreement with a certain problem perception, causal understanding, policy position or implementation of a policy instrument. For instance, one actor could state that nutrient runoff resulting from conventional farming and its entry into waterways threatens the quality of drinking water. Another actor could disagree with this problem perception and would then have an opposed policy core belief.

**Table 2:** Operationalization of actors’ policy beliefs

Policy core beliefs	
Conventional farming threatens drinking water quality	Problem perception
Conventional farming threatens surface water quality	Problem perception
Environmental protection needs organic agriculture	Causal understanding
Tighten Federal Water Act (WHG)	Policy position
Tighten Fertilizer Act (DüG)	Policy position
Tighten Fertilizer Ordinance (DüV)	Policy position
Tighten Fertilizer Regulation (DüMV)	Policy position
Tighten regulation on area designation (AVV GeA)	Policy position
Tighten regulation on farm gate balance (StoffBilV)	Policy position

Secondary aspects	
Limit livestock production to pasture	Policy instrument
Mandatory field-based nutrient accounting	Policy instrument
Prohibit fertilizer application on ecological compensation areas	Policy instrument
Stricter blocking periods for fertilizer application	Policy instrument
Riparian buffer strips	Policy instrument
General upper limit on fertilizer application	Policy instrument
Farm gate balance	Policy instrument
Dung exchange (“Gülle-Börse”)	Policy instrument
Environmental tax on nitrate surplus	Policy instrument
Internal differentiation	Policy instrument

Policy beliefs identified in newspaper articles and press releases

The final list of policy beliefs was identified deductively based on the conceptual definition of policy core beliefs and secondary aspects while reading the documents and coding the data. An iterative coding procedure ensured that every policy belief was coded. The author and two research assistants coded all documents manually with the help of the software *Discourse Network Analyzer* (Leifeld et al., 2019). To ensure intercoder reliability, all three coders coded a sample independently and then compared the coding in order to identify and clarify differences between coders before the final rounds of coding. The final coding entails 2085 statements where actors express their policy beliefs.

Discourse network analysis was then used to analyze coalition formation based on similarity and dissimilarity of actors’ policy beliefs. In a first step, *one-mode adjacency matrices* with actors in rows and columns were derived from the data for the four periods separately. More precisely, these are *one-mode subtract networks,* which combine *congruence networks* and *conflict networks*. Social network analysis uses the term *edge* to indicate the relationship between two actors. In *congruence networks*, two actors are linked with an *edge* (indicated by cell values greater than 0) if they both share at least one belief, i.e., mutual agreement or disagreement with a problem perception or policy position. The more beliefs two actors share, the higher their *edge weight*. In *conflict networks*, two actors are linked with an edge if they have opposing positions regarding at least one belief. The more conflicting beliefs two actors have, the higher their edge weight in the conflict network. The subtract network combines both approaches by subtracting conflict network edges weights from congruence network edge weights (Leifeld, 2017). In this study, the edge weights of the subtract network consider the number of different beliefs two actors share or explicitly do not share. To control for different levels of activity in the debate, the edge weights were normalized using the average-activity algorithm (see Leifeld, 2017, for a detailed discussion on normalization). The final subtract adjacency-matrix contains actors in rows and columns, with cell values ranging from − 1 to 1. Higher values indicate higher belief similarity and lower values indicate higher belief dissimilarity.

Actor coalitions were determined by conducting cluster analyses separately for each period and therefore based on four separate subtract networks. The great advantage of subtract networks is that they consider both similarity and dissimilarity. In these networks, strong polarization is characterized by many positive edges (high similarity) within clusters and many negative edges (high dissimilarity) between clusters (Harrigan et al., 2020; Neal, 2020). This study applied the *Spinglass algorithm* to determine coalitions as it can be used for the analysis of these *signed weighted networks* and has been implemented within the R language (Reichardt et al., 2020). The function identifies clusters characterized by many positive edges and few negative edges within a cluster, and many negative edges and only few positive edges with actors outside the cluster (Reichardt & Bornholdt, 2006; Traag & Bruggeman, 2009). The method is based on the measure of *modularity*, which also serves as an overall indicator of how strongly a network is characterized by cluster formation. Empirically it has been shown that a modularity score larger than 0.3 denotes statistically significant clusters in a network (Leicht & Newman, 2008; Newman, 2004).

The cluster analysis was complemented by a graphical analysis to evaluate the robustness of the results. For this purpose, the four networks were visualized as network graphs by placing the actors as *nodes* in a two-dimensional space based on their similarity using the *Fruchterman–Reingold force-directed placement algorithm.* The algorithm is commonly applied in social network analysis and places groups of nodes, characterized by higher edge weights, closer together. Compared with other placing algorithms, it has the advantage of simultaneously improving the readability of the graph by reducing the overlap of nodes (Fruchterman & Reingold, 1991). As the algorithm can only consider positive edge weights, negative cell values in the subtract adjacency matrices were removed beforehand. Therefore, the approach does not fully incorporate the dissimilarity of actors. Nevertheless, the edge weights still indicate actors’ level of belief similarity controlled by their level of belief dissimilarity. Since actors with higher belief similarity are positioned closer to each other, this graphical approach allows the evaluation of the overall network structure and the identification of actor coalitions (Leifeld, 2013).

Finally, hypotheses 1a, 1b and 1c on differences between coalitions in their participation in the public debate are evaluated based on the number of press releases they disseminate on the policy issue.

### Second part of the empirical analysis: narrative strategies

The analysis of narrative strategies builds on the previous identification of actor coalitions. More specifically, the study used statistical methods to investigate whether the identified coalitions differed in their use of characters and cost–benefit frames and whether they adapted these narrative strategies over time. In addition, discourse network analysis was used to investigate how cohesively coalitions constructed narrative strategies based on their use of characters. The data source for these analyses was restricted to actors’ press releases. The software *discourse network analyzer* was used again to code characters and cost–benefit frames within actors’ press releases based on a codebook (see Appendix B), which was derived deductively from the theoretical approach presented in the theory section.

In this second part of the analysis, the study first investigated whether actor coalitions differed in their use of frames to contain or expand the policy subsystem (hypothesis 2a). Actor coalitions’ use of frames was operationalized by their use of the eight different frames depicted in Table 1 in the theory section. The study used the ratio of containing frames to expanding frames to measure the degree to which a coalition tried to reduce or increase attention to the policy issue. More specifically, a ratio of containing frames to expanding frames with a continuous scale from − 1 to + 1 was calculated where values below zero indicate a predominant use of expanding frames and values above zero the predominance of containing frames. The *contain-expand ratio* was attained by subtracting the sum of expanding frames from the sum of containing frames and dividing the result by the total number of frames used in a press release (the unit of observation). Two-sample t-tests served to explicitly test differences in the use of containing and expanding frames between coalitions. The differences were tested within each period to incorporate the expected time dynamics.

Hypotheses 2b and 2c expect actors to adapt their use of frames over time. To investigate these expected changes, time series of coalitions’ contain-expand ratios were tested for statistically significant trends using *Mann–Kendall Trend Tests*, where a positive trend indicates an increasing level of containment (or decreasing level of expansion) and a negative trend an increasing level of expansion (or decreasing level of containment). The trend test was conducted using aggregated time-series data with mean contain-expand ratios per month.

In a second step, the study analyzed differences and changes in actor coalitions’ use of characters. Hypothesis 3a expects coalitions likely to lose on the policy issue to use the devil shift and those likely to win the angel shift. The *devil shift-angel shift* was operationalized by actors’ use of heroes and villains in their policy narratives. More specifically, the ratio of heroes to villains with a continuous scale from − 1 to + 1 was calculated where values below zero indicate a devil shift and values above zero indicate an angel shift. The ratio was attained by subtracting the number of villains from the number of heroes and dividing the result by the total number of characters used in a press release (Shanahan et al., 2013, 2018). Two-sample t-tests were used to explicitly test differences in the use of heroes to villains between coalitions. The tests used actors’ press releases as units of observation and differences between coalitions are tested for the four periods separately.

Changes in the use of characters over time, as expected by hypotheses 3b and 3c, were investigated by using the same approach already described for analyzing actor coalitions’ use of frames: time series of coalitions’ hero-villain ratio were tested for statistically significant trends using *Mann–Kendall Trend Tests*, where a significant positive trend indicates an increasing angel shift (or decreasing devil shift) and a significant negative trend an increasing devil shift (or decreasing angel shift). The trend test was conducted using aggregated time-series data with mean hero-villain ratios per month.

**Fig. 1:**
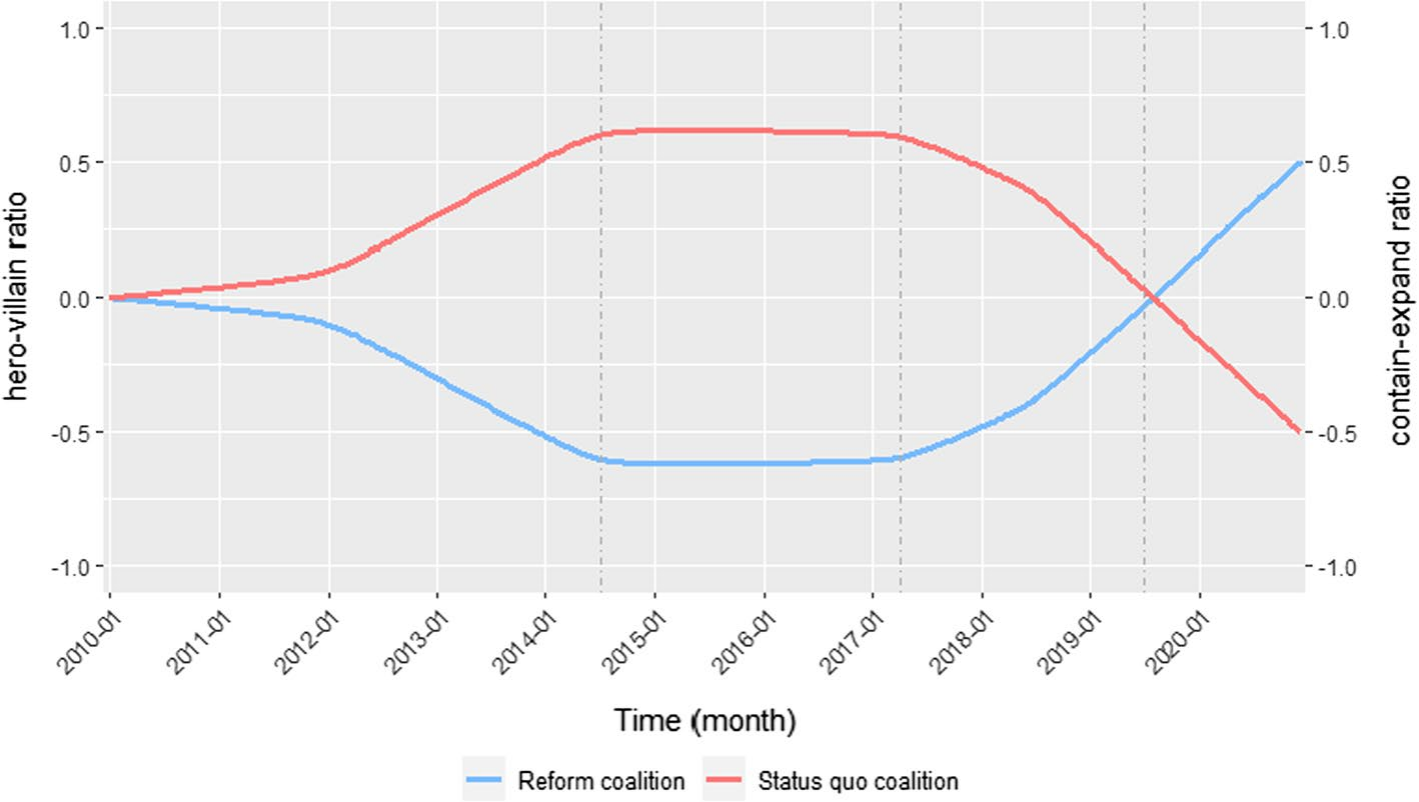
Expected differences and change in coalitions’ use of narrative strategies. Note: The two curves represent approximate curves to visualize the operationalized theoretical expectations on coalitions’ use of frames and characters. The graph is not based on empirical data; the dashed vertical lines subdivide the graph into the four observation periods

Figure 1 visualizes the operationalization of hypotheses 2a to 3c on narrative strategies by drawing approximate curves of the expected contain-expand ratio and the hero-villain ratio. Both ratios are expected to be positive for the status quo coalition and negative for the reform coalition in the first three periods. The positive and negative slope in the first period incorporates the onset of the dispute: the debate was rather technical in the beginning and became more and more politicized, which should be reflected in both coalitions’ documents (from technical reports to policy narratives). With increasing likelihood for the adoption of stricter fertilizer regulation, we can expect a decreasing trend for the status quo coalitions’ ratio of containing to expanding frames and in their ratio of heroes to villains. Simultaneously, we should observe an increasing trend in both ratios for the reform coalition. Theoretically, the curves are expected to cross as soon as the reform coalition perceives a win on the policy issue more likely than the status quo coalition. The ratios should then remain positive for the reform coalition and negative for the status quo coalition as long as the former feels likely to defend the stricter fertilizer regulation and the latter unlikely to liberalize the legal provisions.

**Fig. 2:**
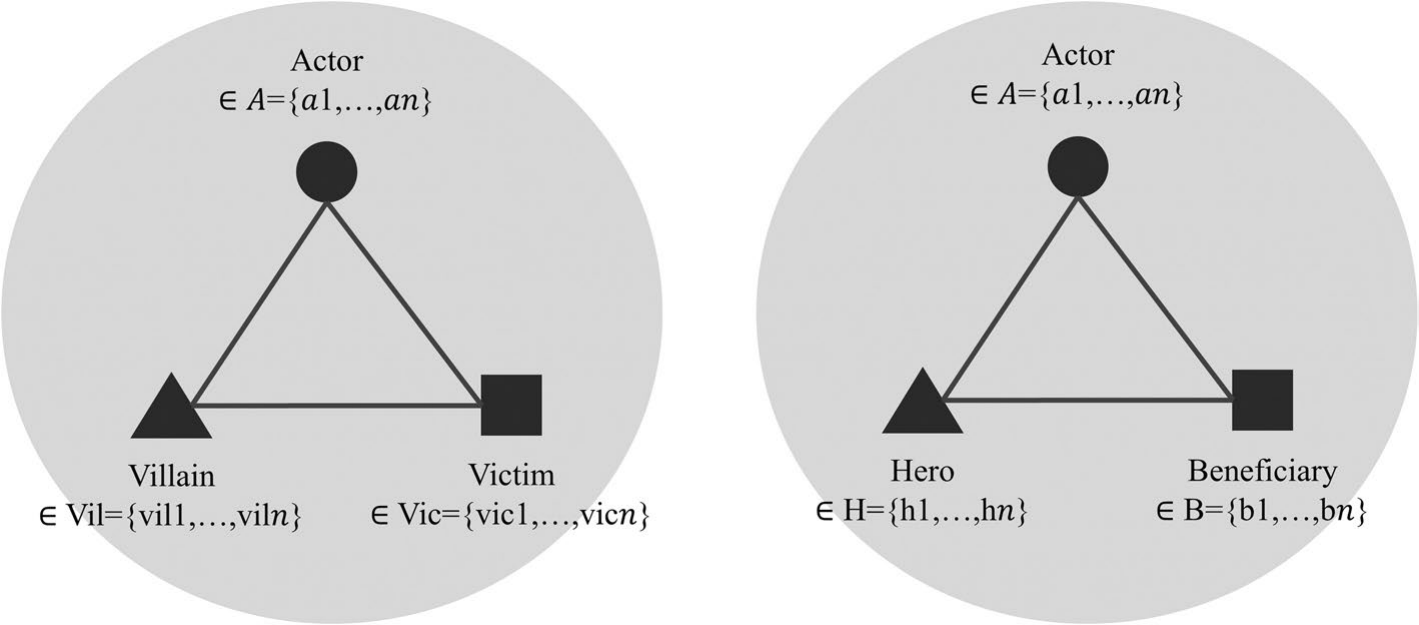
Visualization of character coding

The quantitative analysis of coalitions’ use of characters was complemented by a more qualitative discourse network analysis, which should contribute to an increased understanding of how coalitions deploy characters to expand or contain the scope of conflict. To this end, the coding of characters captured how the actors combined different villains with victims and heroes with beneficiaries (see Fig. 2 for a visualization). Based on the collected data, *bipartite networks* were derived for the coalitions separately to investigate which villainous and heroic causal relationships both coalitions predominantly deployed. A first set of these networks contains villains in rows and victims in columns (villainous) and a second set consists of heroes in rows and beneficiaries in columns (heroic). In all of these networks, two characters are linked by an edge if they were co-referenced by at least one actor. The more often two characters were co-referenced, the higher their edge weight. *Bipartite network graphs* visualize the use of villainous and heroic relationships for both coalitions separately.

To check whether members within coalitions differed in their use of characters, the final part of the analysis investigated *one-mode congruence networks* derived separately for both coalitions, where actors are linked by an edge if they used at least one identical character. The more often two actors deployed identical characters in their policy narratives, the higher their edge weight. Thus, the edge weights not only incorporate the number of identical characters two actors used within a period, but also how frequently they co-referenced identical characters. The final edge weights were normalized by average activity to control for different levels of activity. Computation of the networks’ *density* and *modularity* allows for evaluating how cohesive coalitions are in their use of characters. Density is a measure used to capture how strongly connected actors are in a network. It is obtained by dividing the number of edges present in a network by the maximum possible number of edges in a network. The higher the density, the more connected a network is, and in this case, the more cohesive a coalition is in its use of characters. Since the density measure does not consider edge weights, the networks’ modularity was computed as well. Cohesive coalitions are characterized by the absence of any clusters of actors within the coalition. Thus, a low modularity score can be interpreted as an indication of high cohesiveness.

## Results

This section first reports the findings on the coalition formation within the policy subsystem (hypotheses 1a–1c) and then presents the results of the analyses of actor coalitions’ use of narrative strategies (hypotheses 2a–3c).

### Coalition formation and participation in the public debate on fertilizer regulation

**Fig. 3:**
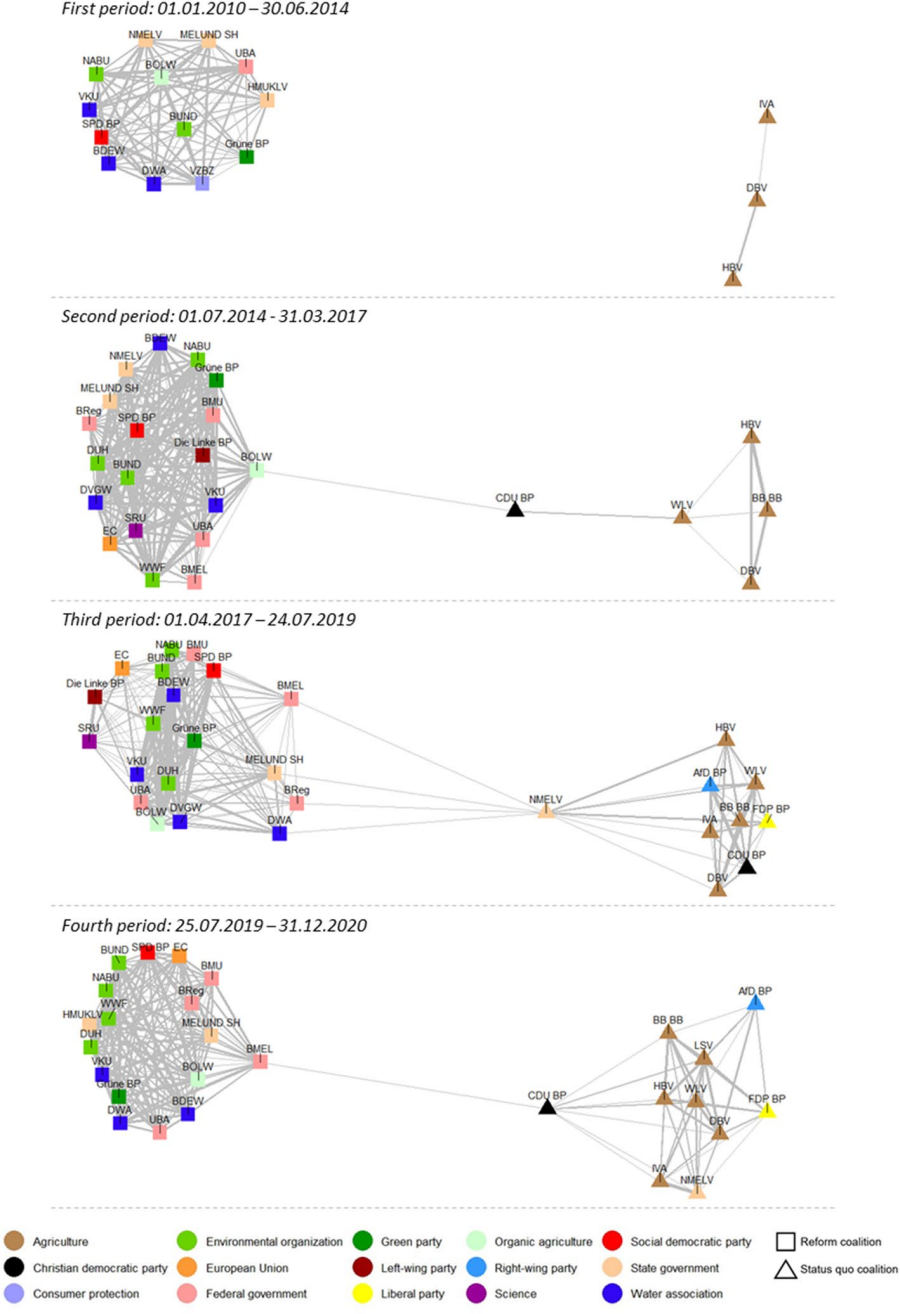
Actor coalitions determined through discourse network analysis. Note: The graph shows subtract actor networks normalized by average activity. See Table S1 for full list of actors

The results of the discourse network analysis on coalition formation show that the German public debate on agricultural nitrate pollution of freshwater between 2010 and 2020 was characterized by increasing polarization over time and the formation of two adversarial actor coalitions. Figure 3 visualizes the actor networks in each period by plotting the actors as nodes and their degree of similarity as edges in a two-dimensional space. The thicker the edges, the greater two actors’ similarity. Node colors indicate an actor’s affiliation, such as environmental or agricultural organization. The shape of nodes (rectangles and triangles) visualizes the results of the cluster analysis.

Figure 3 shows that actors cluster into two densely connected groups with only a few similarity edges between the clusters in all of the four periods. The visually identified groups concur with those determined by the cluster analysis. The group plotted on the left-hand side in each graph was in favor of stricter regulation on fertilizer use in agriculture and will subsequently be termed the *reform coalition.* The coalition mostly consisted of environmental organizations, water associations and the Green party. In addition, some governmental actors at the state, federal and European level as well as center-left and leftist parties joined the coalition over time. The group plotted on the right-hand side opposed stricter regulation and will be referred to as the *status quo coalition.* The coalition predominantly consisted of farmer associations. Interestingly, the coalition grew over time: new established farmer associations, such as the *LSV* or *Freie Bauern* (former *BB BB*) and political parties including the *CDU*, *FDP*, and *AfD* join the coalition*.* The first two parties are no surprise since the CDU has always been farmers’ main political representative and the neo-liberal FDP usually opposes stricter state regulation (Tosun, 2017). The AfD joined farmers’ side to win the votes of frustrated former CDU and CSU supporting farmers (FAZ, 2020a, b).

Both coalitions were mostly congruent in their beliefs with only a few deviations (see Table S1 and Table S3 in Appendix A for a detailed overview on actors’ articulated beliefs). The deviations mostly concern governmental actors. The BMEL plays a special role here since it is responsible for drafting regulation on the issue and was led by CSU and CDU between 2010 and 2020. On the one hand, the BMEL has always leaned toward farmers’ interests when led by one of these two parties since farmers represent an important part of their electorate (Tosun, 2017). On the other hand, increasing pressure by the EC to tighten regulation on fertilizers seems to have affected the BMEL’s position on the issue. The ministry further tended to take ambiguous positions in the second half of the observation period, where it appeared as a *policy broker* rather than a member of any of the two coalitions. Research on policy brokerage has shown that these actors are distinct from other actors. They tend to moderate between adversarial coalitions with an interest in reaching feasible policy outputs (Christopoulos & Ingold, 2015).

The results of the discourse network analysis further indicate that the policy subsystem was characterized by increasing polarization. First, the computed modularity score for the whole network increases over time (0.17, 0.18, 0.31, 0.37). Second, actors mostly stayed within their coalition. The state ministry for agriculture of Lower Saxony (NMELV) was the only exception. The state ministry changed sides in the third period, which can be explained by a change in government in Lower Saxony in November 2017. The state ministry had been in charge by the Greens and was then led by the CDU after the election.

**Fig. 4:**
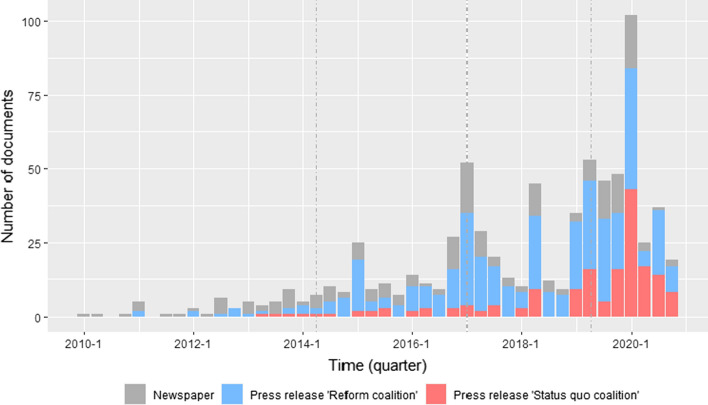
Change in salience of the policy issue indicated by news coverage and the number of press releases published by the actor coalitions. Note: The vertical dash lines indicate the end of each period

The identified coalitions differed in how strongly they participated within the debate. Figure 4 reports the number of newspaper articles and press releases published by both coalitions over time. Press releases published by the reform coalition increased over time until March 2020 and clearly outnumbered those of the status quo coalition (383 to 171 press releases). Both, the higher number of press releases published by the reform coalition and the larger number of actors present in the debate at the onset of the conflict provide support for hypothesis 1a, which expected the coalition challenging the status quo to resort to the arena of public debate more strongly.

The number of press releases published by the status quo coalition increased significantly with the start of the fourth period in July 2019, and even outnumbered those of the reform coalition in the first half of 2020. Figure 3 already shows that the status quo coalition grew in members over time. Both observations support hypothesis 1b, which expected the status quo coalition to increase its level of participation in the public debate with increasing likelihood to lose on the policy issue. However, the pattern for the reform coalition is not as clear. Although their number of press releases decreased in the fourth period, the coalition still participated actively in the debate. Thus, support for hypothesis 1c is only limited, which expected coalitions to reduce their level of participation in the public debate in response to an increase in the likelihood to win on the policy issue.

To summarize, two adversarial coalitions formed in the public dispute on agricultural nitrate pollution of water, who mobilize for and against stricter fertilizer regulation. The various political actors can be assigned clearly to either of the two coalitions, except for governmental actors who tended to be less consistent in the articulation of their policy beliefs. The status quo coalition participated less actively at the onset of the dispute, which confirms hypothesis 1a. In line with hypothesis 1b, the status quo coalition then increasingly resorted to the public debate with increasing likelihood to lose on the issue. The reform coalition reduced its level of participation in the debate only moderately despite the increased likelihood to win, which supports hypothesis 1c only to some extent.

### Narrative strategies

The second empirical part of this study investigates the narrative strategies employed by the previously identified coalitions. By doing so, it only investigates the narrative strategies of non-governmental actors of both coalitions. The decision to exclude governmental actors (such as the EC, federal or state ministries) has been made based on the findings of a first preliminary empirical analysis, which suggested that governmental actors behave differently regarding their narrative strategies. In contrast to non-governmental actors, the results suggested that governmental actors tend to contain the scope of conflict, by predominantly using containing frames and heroic causal relationships, independent of their coalitional membership, i.e., their policy beliefs and preferred policy outcome. One reason could be their responsibility for dealing with the policy issue and, therefore, an incentive to narrate a plot of control, where they overemphasize their role as successful problem-solvers, diffuse their actions’ benefits and concentrate any associated costs, to cast a good light on their own actions. In addition, results of the analysis on coalition formation already suggested that governmental actors tend to articulate their policy beliefs less consistently and might pursue different aims, such as reaching achievable policy compromises. Finally, the decision is substantiated by a recent contribution by Weible et al. (2020) who argue for a differentiation between distinct types of coalition members. In this sense, the study focuses on the behavior of principal coalition members in the subsequent analysis.

#### Cost–benefit frames used to contain or expand the scope of conflict

The first part of the analysis of coalitions’ narrative strategies investigates their use of cost–benefit frames to either contain or expand the scope of conflict. Hypotheses 2a–2c expect coalitions to differ in their use of frames dependent on the likelihood to win on the policy issue, where a likelihood to win is associated with containing frames and a likelihood to lose with expanding frames. Table 3 gives an initial overview on coalitions’ use of frames by reporting their absolute number and relative frequency for both coalitions separately for the whole observation period.

**Table 3:** Cost–benefit frames used by the two coalitions

	Status quo coalition		Reform coalition	
	Ʃ	%	Ʃ	%
Diffuse costs of stricter regulation	70	72.2	0	0
Concentrate benefits of stricter regulation	3	3.1	0	0
Diffuse benefits of liberalization	1	1.0	0	0
Concentrate costs of liberalization	23	23.7	0	0
Diffuse costs of liberalization	0	0	194	86.6
Concentrate benefits of liberalization	0	0	5	2.2
Diffuse benefits of stricter regulation	0	0	20	8.9
Concentrate costs of stricter regulation	0	0	5	2.2
Sum	*97*	*100*	*224*	*100*

The table reports the absolute number of frames and their relative frequency for both coalitions separately

In general, both coalitions used frames according to their preferred policy outcome (liberalization vs. stricter regulation). Members of the status quo coalition diffused the costs of stricter regulation (72.2% percent of their frames) and concentrated its benefits (3.1%). For instance, the LSV diffused the costs of stricter fertilizer regulation to the German population when it wrote in March 2020 at the onset of the COVID-19 pandemic: “The revision of the fertilizer ordinance must be stopped. It would lead to a situation where farmers can no longer guarantee basic food supplies.” The Freie Bauern concentrated the benefits of stricter regulation by stating in May 2020: “Svenja Schulze […] is part of the Federal Government, which systematically disadvantages domestic agriculture for the benefit of industrial export interests.” On the other hand, the status quo coalition diffused the benefits of liberalization (1%) and concentrated its costs (23.7%). An example for the diffusion of benefits of liberalization is the following statement by the DBV in January 2015: “The ‘sweeping demonization’ of nitrogen fertilization is not helpful. Nitrogen makes an important contribution to world nutrition.” The predominant way in which the coalition downplayed the costs of liberalization was to narrow nitrate pollution down to few geographical areas. For instance, the Freie Bauern stated in August 2019: “Tightening fertilizer regulation is not necessary for more than 95 percent of German agricultural land. Only a few selected regions with high livestock density need to be looked at more closely.”

Members of the reform coalition diffused the costs of liberalization (86.6%) and concentrated its benefits (2.2%). For instance, Alliance ‘90/The Greens diffused the costs of nitrate pollution by writing in August 2017: “According to a study by the UBA, nitrate concentration limits in drinking water are often only achieved through costly water treatment. Ensuring clean drinking water involves costs to society of up to 25 billion euros per year. If we do not counteract nitrate pollution, we will all pay the price.” An example for concentrating the benefits of liberalization was also provided by the Green Party, which stated in July 2018: “The interest of factory farmers may not have higher priority than water protection.” On the other hand, the reform coalition diffused the benefits of stricter regulation (8.9%) and concentrated its costs (2.2%). An example for the diffusion of the benefits of stricter regulation was provided by the SPD in March 2020: “Today, the Bundesrat approved the necessary revision of the fertilizer ordinance […], and, thereby, set the course for the sustainable supply of clean drinking water.” The DUH concentrated the costs of stricter fertilizer regulation by stating in June 2020: “Implementing the new fertilizer legislation will cost German agriculture only a fraction of the penalty Germany would have had to pay in case of a second EU infringement procedure.”

Table 3 shows that both coalitions predominantly used expanding frames (status quo coalition: 75.3%; reform coalition: 88.8%) and substantially less frequently containing frames (24.7% and 12.2%). With this in mind, the subsequent analyses show that differences between coalitions and changes over time are mostly related to how strongly both coalitions used expanding frames in their press releases.

**Fig. 5:**
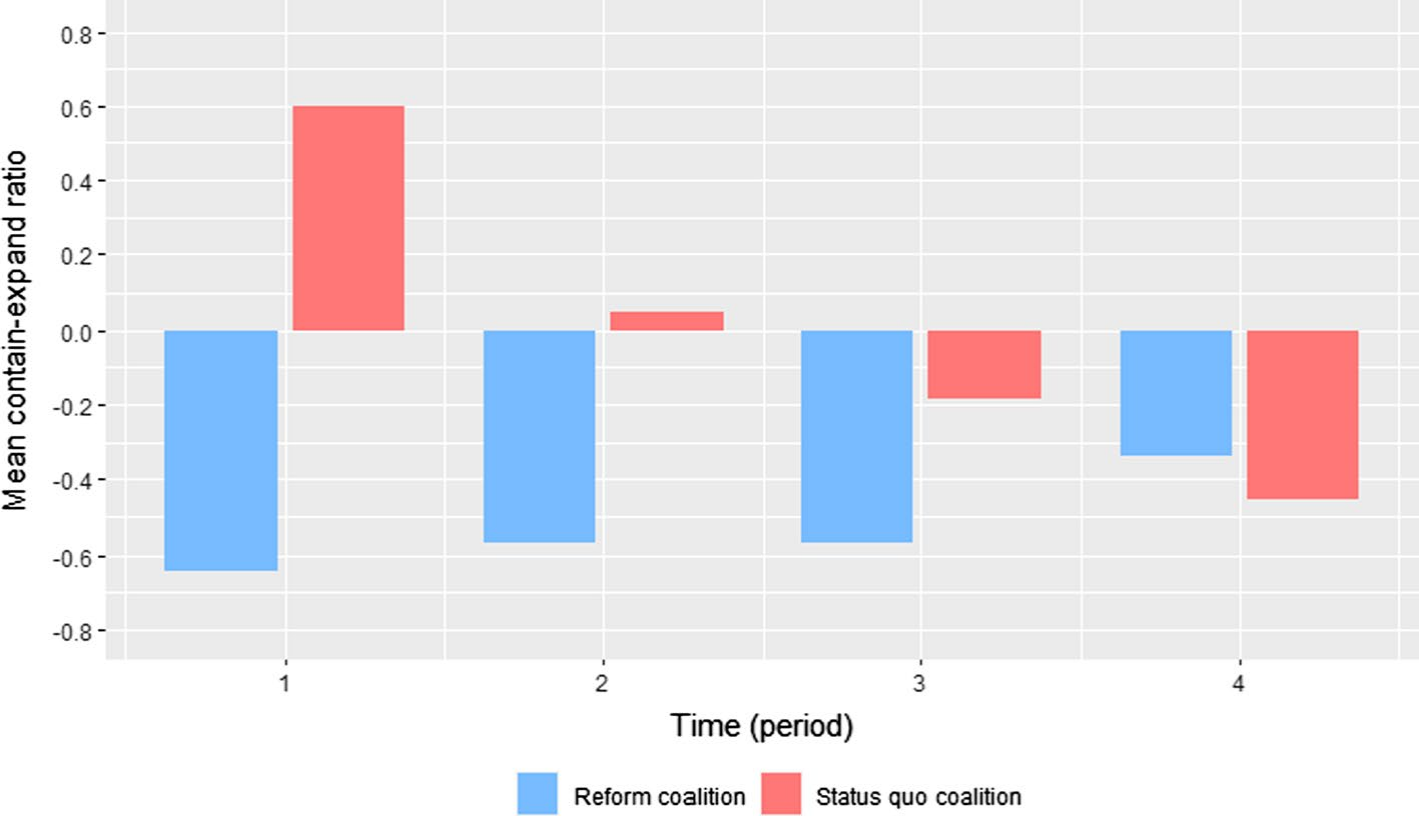
Coalitions’ use of expanding and containing frames in each period. Note: The graph reports mean ratios of containing frames to expanding frames within documents per period, plotted for both coalitions separately

The analysis of coalitions’ use of frames mostly support the theoretical expectations of hypotheses 2a, 2b and 2c. Both coalitions differed in their ratio of containing to expanding frames and adapted their use of frames in response to changes in the likelihood to win or lose. Figure 5 plots the mean ratio of containing frames to expanding frames within actors’ press releases in each period for the two coalitions separately. As expected by hypothesis 2a, the reform coalition’s narratives were more strongly characterized by expanding than containing frames at the onset of the conflict, which is indicated by the negative ratio in the first period. Over time, the reform coalition increased the ratio of containing frames to expanding frames, which is in line with the expectation of hypothesis 2c on changes in response to an increasing likelihood of winning. However, the reform coalition continued to use more expanding than containing frames in the fourth period, despite the change in likelihood to win on the issue. This is not in line with hypothesis 2a, which expected the reform coalition to predominantly use containing frames as soon as it is more likely to win on the issue.

The status quo coalition used more containing than expanding frames in their press releases at the onset of the dispute, as expected by hypothesis 2a. Over time, the coalition reduced the ratio of containing frames to expanding frames, which supports the expectation of hypothesis 2b on changes in response to an increasing likelihood of losing. In the end, the status quo coalition used more expanding than containing frames, as expected by hypothesis 2a since the coalition is then more likely to lose on the issue.

The results of the conducted t-tests mostly confirm the observed differences between coalitions (see Table S6 for details on the test results). In the first period, the status quo coalition had a statistically significant higher ratio of containing frames to expanding frames compared to the reform coalition (*p* < 0.01). The difference in means remained statistically significant in the second and third period (both *p* < 0.01). Only the small difference in the fourth period is not statistically significant (*p* > 0.1).

**Fig. 6:**
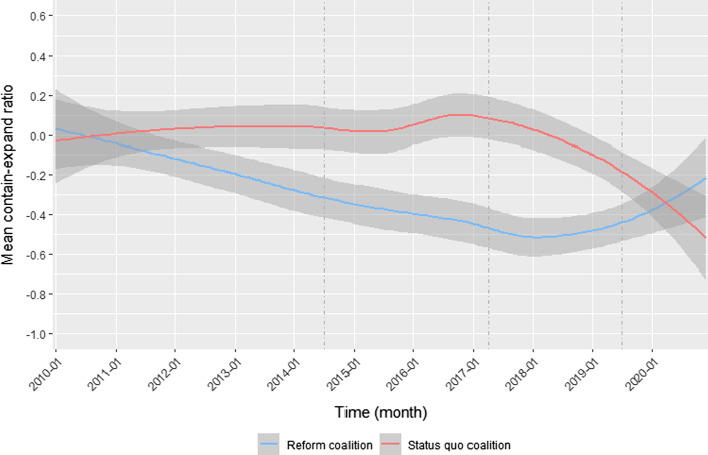
Changes in coalitions’ use of cost–benefit frames over time. Note: The graph reports smoothing lines estimated by using the non-parametric LOESS (locally estimated scatterplot smoothing) method based on mean ratios of containing to expanding frames within documents per month. The gray shaded areas around the lines represent 90% confidence intervals

Figure 6 gives a more detailed insight into coalitions’ use of frames over time. More specifically, it plots smoothing lines for each coalition based on the ratio of containing frames to expanding frames per document aggregated by month. The smoothing lines were estimated by applying the non-parametric LOESS (locally estimated scatterplot smoothing) method, which is commonly used to find a curve of best fit in time-series data. The graph already shows that the empirically estimated lines resemble the theoretically derived and approximately drawn curves in Fig. 1 quite well. The two lines diverge in the first period and then cross in the fourth period.

Results of the Mann–Kendall–Trend Tests based on the monthly time-series data confirm the observed changes in frame use over time for both coalitions and substantiate the findings on hypotheses 2b and 2c (see Table S7 in Appendix C for detailed results). Regarding the reform coalition, the trend test suggests a statistically significant negative trend in the first two periods (*p* < 0.01). The coalition’s use of frames changed in the third and fourth period, indicated by an increase in the ratio of containing frames to expanding frames (*p* < 0.1). In fact, the coalition reduced their use of expanding frames per document in these periods (see Figure S1 in Appendix C) whereas the level of containing frames remained about equal (see Figure S3 in Appendix C). The turn in the third period aligns well with hypothesis 2c. The first ruling by the CJEU in June 2018, which found Germany to be in breach of its obligations in implementing the nitrate directive, and the official warning letter sent from the European Commission in July 2019 increased the likelihood for stricter regulation significantly. In accordance with these events and the increased likelihood to win on this policy issue, members of the reform coalition decreased their efforts to expand the policy issue.

Regarding the status quo coalition, Fig. 6 suggests that the ratio of containing frames to expanding frames remained constant around 0 in the first two periods, indicating no clear tendency toward containment or expansion. The Mann–Kendall Trend Test confirms the absence of a negative or positive trend in these first two periods (*p* > 0.1). This is not in line with hypothesis 2a and the previous observation of a strong use of containing frames in the first period. One reason for the deviation could be the low number of observations in this period (only five press releases for the status quo coalition). Nevertheless, there is support for hypothesis 2b: the smoothing line in Fig. 6 points to a turn in the coalition’s use of frames already at the end of the second period, followed by a negative trend in the ratio (*p* < 0.01). The decrease in the ratio of containing frames to expanding frames in the last two periods is both due to an increase in the use of expanding (see Figure S1 in Appendix C) and a decrease in the use of containing frames per document (see Figure S3 in Appendix C). Thus, the status quo coalition responded to an increasing likelihood to lose on the policy issue as expected by hypothesis 2b.

To summarize, there is support for hypotheses 2b and 2c: both the coalition mobilizing for stricter regulation and the coalition advocating liberalization adapted their use of frames in accordance with the likelihood to win or lose on the policy issue. Evidence for hypothesis 2a is mixed: both coalitions did not predominantly use containing frames during phases in which they were expected to perceive a higher chance of winning. Rather, it appears that both coalitions differed in how strongly they resorted to expanding frames dependent on how likely they were to win or lose on the issue.

#### Use of characters to contain or expand the scope of conflict

**Fig. 7:**
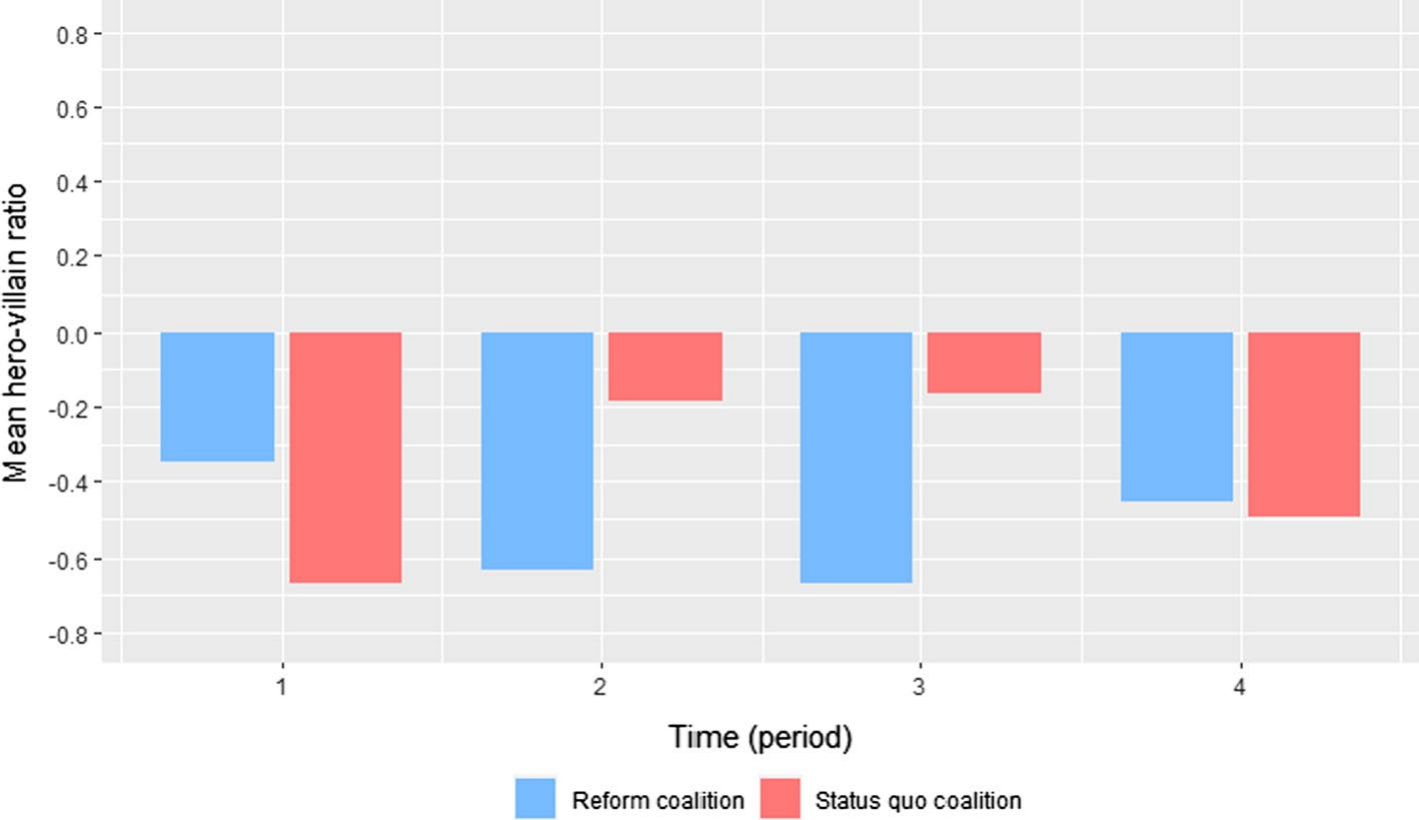
Coalitions’ use of heroes and villains in each period. The graph reports mean ratios of heroes to villains within documents per period, plotted for both coalitions separately

The second part of the analysis of coalitions’ narrative strategies investigates their use of villains and heroes to either contain or expand the scope of conflict. Figure 7 plots the mean ratio of heroes to villains within actors’ press releases in each period for the two coalitions separately.

The policy narratives were generally characterized by a devil shift: both coalitions predominantly used villains in all periods. This is contrary to hypothesis 3a, which expected the coalition with a higher likelihood of winning to use predominantly heroic causal relationships (angel shift). Similar to the cost–benefit frames, coalitions rather differed in how strongly they populated their narratives with villains, dependent on how likely they were to win on the issue (see also Figure S6 and Figure S8 in Appendix D for differences in mean numbers of villains and heroes per period). In general, both coalitions used heroes only rarely compared to villains (see Figure S7 and Figure S9 in Appendix D for absolute numbers of villains and heroes in each period).

When looking at the differences between coalitions, the mean hero-villain ratios observed in the last three periods are in principle in line with hypothesis 3a: The reform coalition resorted more strongly to villains in the second and third period compared to the status quo coalition, which corresponds with its lower likelihood to win on the issue at this time. The observed differences in means are statistically significant (*p* < 0.01) in these two periods (see Table S8 in Appendix D for full results). In accordance with the higher likelihood to lose on the policy issue in the fourth period, the status quo coalitions’ policy narratives became more villainous. In contrast, those of the reform coalition changed to being less villainous compared to the previous period. Consequently, both ratios converged with no statistically significant differences (*p* < 0.1). Only the first period does not fit the expectations, where the observed difference in this period is counter-intuitive: the status quo coalition should have been more likely to win and, therefore, less frequently blame its opponents in their narratives than the reform coalition. However, the number of observations in this period is small (as we know already from the analysis of cost–benefit frames) and the t-test is not statistically significant (*p* > 0.1). Thus, the observed difference is likely a result of random chance.

**Fig. 8:**
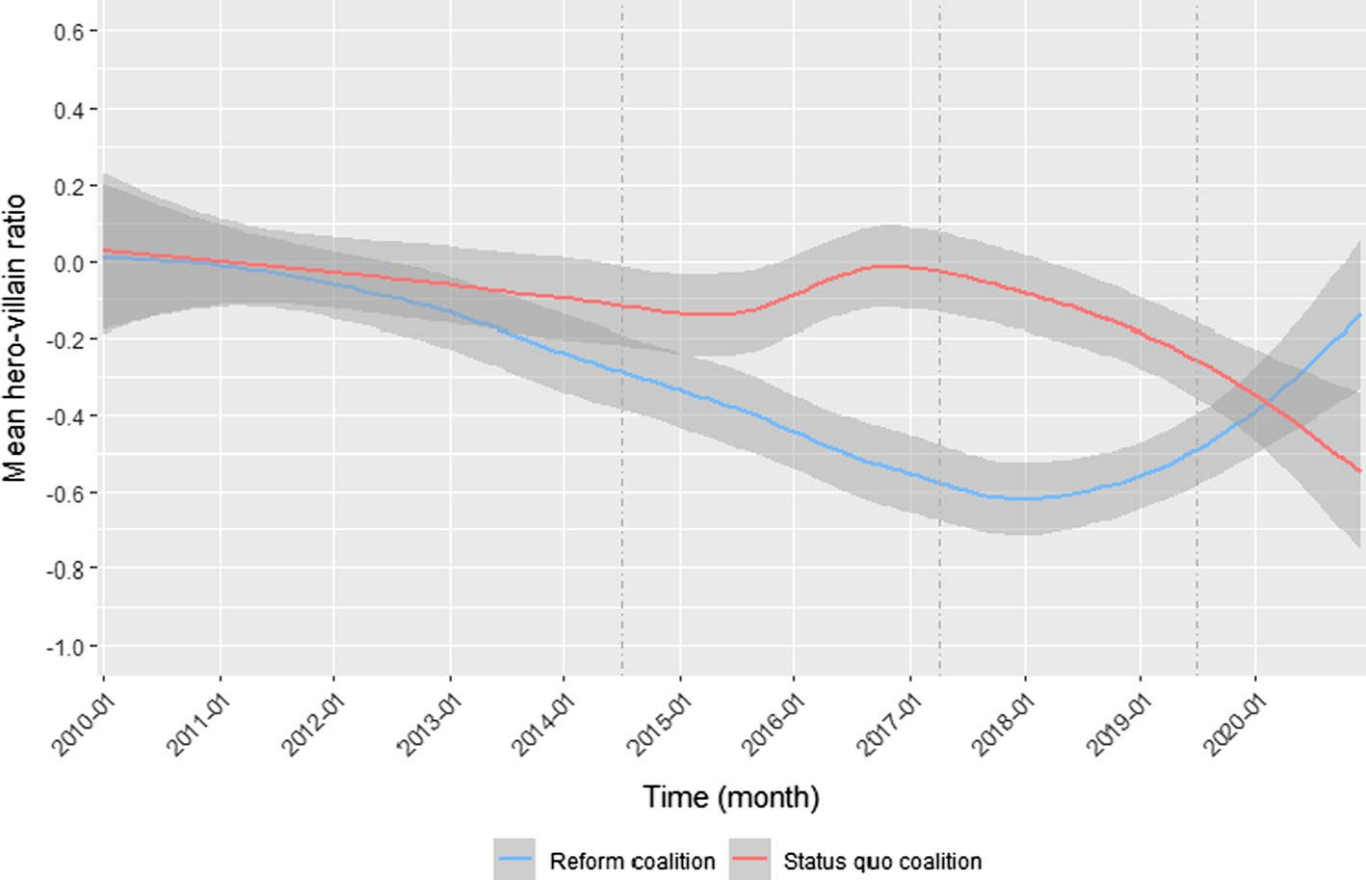
Changes in coalitions’ use of characters over time. Note: The graph reports smoothing lines estimated by using the non-parametric LOESS (locally estimated scatterplot smoothing) method based on mean hero-villain ratios within documents per month. The gray shaded areas around the lines represent 90% confidence intervals

Figure 8 provides more detailed insight into coalitions’ use of characters over time by plotting smoothing lines for each coalition based on the monthly mean ratio of heroes to villains, similar to Fig. 6 in the analysis of cost–benefit frames.

Results of the Mann–Kendall Trend Tests on the monthly data mostly confirm the changes in coalitions’ use of villains and heroes over time as expected by hypotheses 3b and 3c (see Table S9 in Appendix D for full results). When looking at the reform coalition separately, Fig. 8 shows a negative trend in the hero-villain ratio in the first two periods, which is confirmed by the trend test (*p* < 0.01). The reform coalition used more villains than heroes pointing toward an increasing level of antagonism, which is aligned with the coalition’s lower likelihood to win on the issue. Members of the coalition were especially dissatisfied with the revision of the fertilizer ordinance in March 2017 and consequently disseminated many villainous press releases around this time. The reform coalition then changed its behavior in the third period. From 2018, the trend became positive indicating decreasing levels of antagonism over time (*p* < 0.01). At that time, the coalition’s likelihood to win on the issue increased considerably through the CJEU’s decision against Germany and later through the EC’s threat to open a second infringement procedure. Thus, this change in the reform coalition’s use of characters aligns well with hypothesis 3c and coincides with its change in the use of cost–benefit frames at the same time as observed before.

Regarding the status quo coalition, Fig. 8 also suggests a negative trend in the first period, followed by an upward trend in the second period. The results of the Mann–Kendall Trend Test point toward a negative trend within the first two periods (*p* < 0.05), which is not in line with the theoretical expectations. This finding rather indicates an increasing level of antagonism for the status quo coalition simultaneous to an increasing level of polarization of the debate. Complementary to the change in the reform coalition’s behavior, the Mann–Kendall Trend points toward a proceeding negative in trend in the status quo coalition’s hero-villain ratio within the last two periods (*p* < 0.01). Thus, the status quo coalition disseminated increasingly villainous policy narratives in response to increasing likelihood to lose on the issue, which aligns well with hypothesis 3b.

To summarize, coalitions’ use of characters was similar to their use of cost–benefit frames. Both coalitions predominantly used villains and only occasionally heroes, i.e., they only resorted to the devil shift. Thus, there is no support for hypothesis 3a regarding its expectation on the angel shift. Nevertheless, the results suggest that coalitions differed in how strongly they used the devil shift depending on the likelihood to lose or win on the issue. In addition, both coalitions adapted their use of characters over time, which supports hypotheses 3b and 3c.

##### Qualitative analysis of coalitions’ use of characters

Complementary to the quantitative analysis of coalitions’ use of characters, this part of the empirical analysis sheds light on how coalitions differed qualitatively in their use of characters. It focuses on the villain-victim relationships due to the low occurrence of heroes and beneficiaries (for the sake of completeness, see Figure S11 and Figure S12 in Appendix D for a visualization of hero-beneficiary relationships). The analysis of these relations is insightful since it shows how the two coalitions try to win different target groups’ favor in their aim to expand the scope of conflict.

**Fig. 9:**
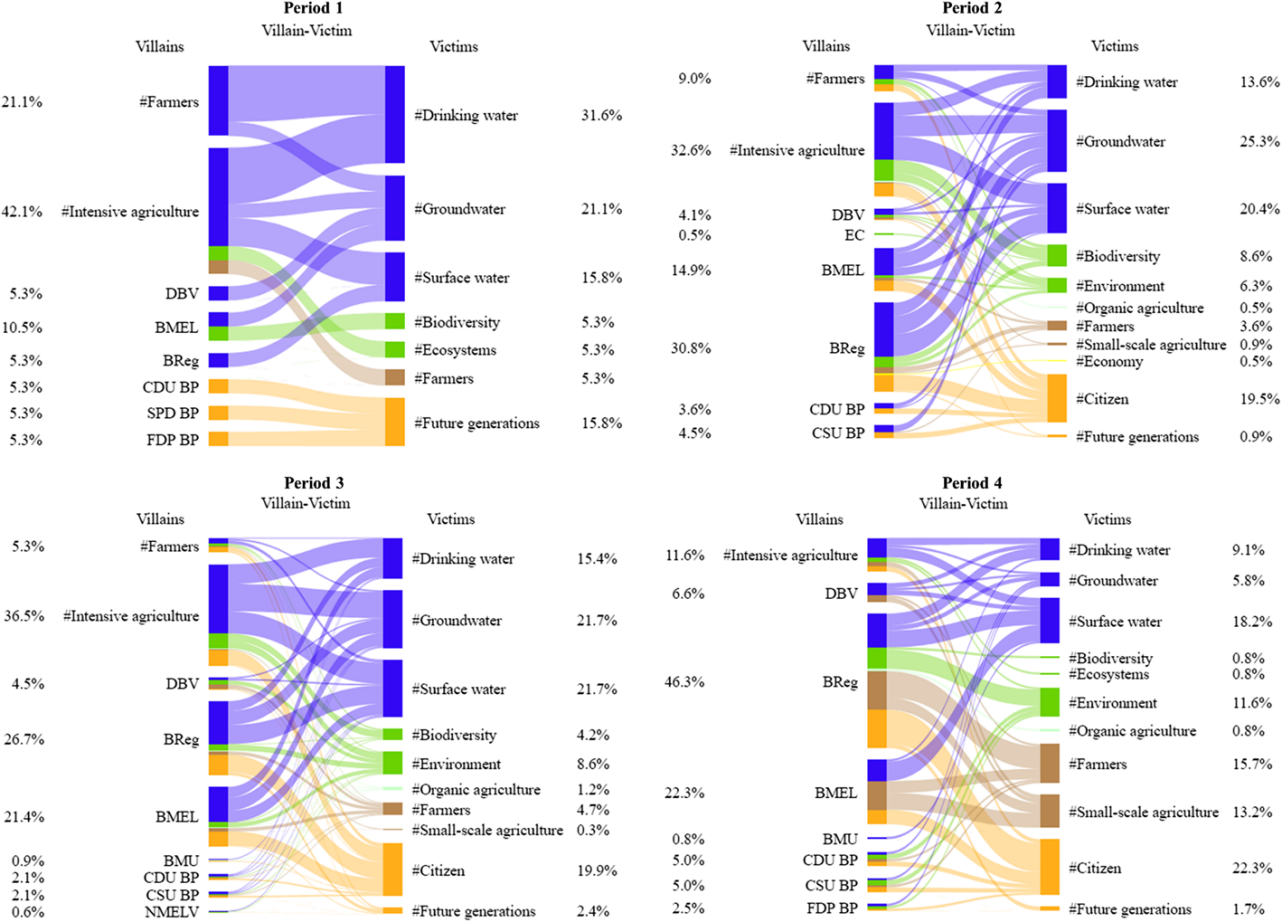
Use of villains and victims by the reform coalition. Note: See Table S1 for full list of actors

**Fig. 10:**
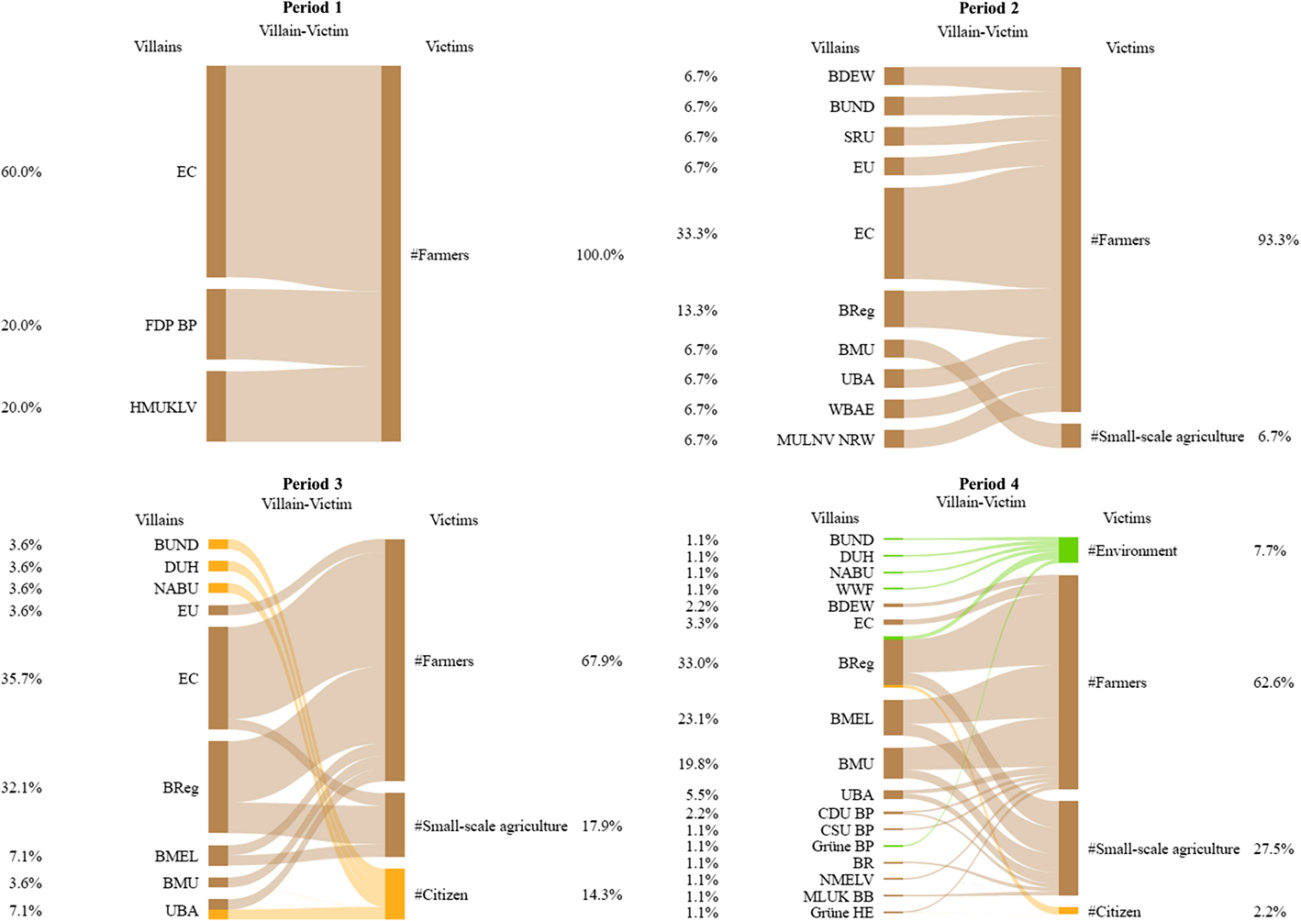
Use of villains and victims by the status quo coalition. See Table S1 for full list of actors

Figures 9 and 10 show bipartite network graphs for each period to visualize how the two coalitions used villains and victims in their narratives to blame opponents and emphasize how they harm various target groups.^4^ Percentages reported on the left-hand side of each graph denote how frequently a coalition named a certain actor or category as a villain. Analogously, percentages on the right-hand side of each graph report the frequency of named victims. The size of the interacting area between a villain and victim pair indicates the frequency of their combination. To give one example on how to read the figures, the graph in the upper-left corner in Fig. 9 shows that in the first period members of the reform coalition most frequently blamed *intensive agriculture* for causing harm (42.1%) to *drinking water* resources, *surface water*, *groundwater*, *ecosystems,* and *farmers*.^5^ On the other hand, the reform coalition most frequently pointed to harm caused to *drinking water* resources (31.6%) by *intensive agriculture* and *farmers* in general. The bars and interacting areas are colored by the type of the associated victim.

When looking at the reform coalition’s use of villainous causal relationships in Fig. 9, it is apparent that its members addressed two specific target groups to increase attention on the issue: citizens and farmers. Gaining stronger citizen support can be influential, either directly through a larger vote share in an upcoming election or indirectly through public opinion, which may influence the behavior of decision makers (McCombs & Valenzuela, 2021; Soroka & Wlezien, 2009). One way coalition members tried to win citizen attention was highlighting the risks posed to drinking water resources and associated health issues. For instance, the Greens stated in January 2019: “The protection of groundwater and, thus, human health, needs highest priority. The inaction by Julia Klöckner is irresponsible.”^6^ Another way was to highlight the financial burden taxpayers carry for securing high quality drinking water and the costs they would have had to pay in case of a second decision by the CJEU against Germany. For instance, the BUND stated in October 2014: “Overuse of fertilizers in intensive agriculture already results in up to 25 billion euros yearly to secure clean drinking water. These external costs produced by industrialized agriculture are currently not paid by the polluter but by the consumer. There will be millions of Euros of additional penalty payments to be paid to the EU if the federal government and the states continue breaching European water protection law.” A third way to address citizens was to stress the importance for future generations, which made the issue personally relevant for many citizens (many have children, will have grandchildren, etc.). The BDEW, for example, mobilized for a significant change in fertilizer regulation in March 2019 by stating: “Every approach, which only aims at repair in waterworks, is at the expense of future generations. For this reason, we need effective fertilizer legislation and strict monitoring of its compliance.”

Apart from citizens, members of the reform coalition appealed to farmers in small-scale agriculture. At first, this seems counter-intuitive. However, members of the reform coalition, e.g., the Green Party, argued that farmers’ livelihoods were not necessarily threatened by stricter fertilizer regulation, but by the sudden need to adapt their farming practices to new regulations, which, in their view, was only caused by the continued inaction of the federal government to transpose the ND into domestic law. For instance, the Green party declared in June 2018: “The federal government has given in to the insinuations by the agricultural lobby represented by the DBV and the Union [CDU and CSU] for years and nothing has happened. Instead, the federal government has been putting off the problems and has even ignored scientific advice on how to improve fertilizer regulations. Now the consequences of this mistaken policy become apparent: Taxpayers and those farms, who contributed to the protection of the environment and groundwater through professional and responsible practice, are now paying the price.” Farmers mentioned in this statement included those in organic agriculture. Thus, the Green party was joined by the BÖLW, Germany’s main representative of organic agriculture. The organization stated in January 2020: “We are sick of the ministry of agriculture’s tokenism! The federal government is responsible for the death of farms [Höfesterben] and the frustration in rural areas. 130.000 farms had to close down since 2005 when Angela Merkel became chancellor – this is on average one family farm per hour.”

When looking at the portrayal of villains, the reform coalition mostly conveyed a clear picture of whom it regarded responsible for the harm caused to citizens, farmers, water quality and the environment: intensive agriculture and the DBV on the one hand and the influence of CDU and CSU in the federal government, especially in the BMEL, on the other hand. This was mostly consistent over the four periods.

Finally, the reform coalition was also very cohesive in how it blamed villains for harming victims. Modularity in the derived one-mode congruence networks is very low with a modularity score around zero in all periods suggesting no significant differences between members of the coalition (see Table S10 for detailed results). High levels of density support the impression of high cohesiveness. The density levels are a bit lower in the first and fourth period, which is mostly due to outliers who only rarely used villains or victims in their press releases and share no edges with any other members of the coalition.

Figure 10 visualizes the use of villains and victims by the status quo coalition. The frequencies reported in the first two periods need to be interpreted with caution due to a comparatively small number of villains and victims in these two periods.

Overall, members of the status quo coalition mostly focused on farmers when portraying the harm caused to victims by villains. Since farmer associations represented most members of the status quo coalition, this is not surprising. Nevertheless, there is an interesting change over time: the coalition increasingly focused on small-scale agriculture (from 0 to 27.5%). The change is partly due to *LSV*, who formed only in October 2019 as a new organization to represent the interests of small-scale farmers, and the move of *BB BB* to become the nationwide organization *Freie Bauern*, which also is a representative of small-scale farmer interests. In addition, political parties jumped on the bandwagon. One of them was the *FDP*. Agriculture and water protection do not represent key policy fields for the party, which is also indicated by no published press releases on the issue of nitrate pollution before 2019. In October 2019, however, the *FDP* wrote in a press release: “We support the reasonable protests of thousands of farmers. They show how the agricultural policy of both federal ministers […] threatens to deprive a whole branch of their means of existence and leads to unfair competitive conditions for German agriculture.” The AfD was the second party suddenly starting to mobilize for the interests of small-peasant farms at the end of 2018. For instance, they stated in a press release in January 2020: “Especially part-time farmers and the small family farms will not be able to compensate the additional costs resulting from the stricter fertilizer ordinance. There is the threat of a massive structural upheaval, which would be followed by a tremendous concentration process in agriculture. The AfD federal parliamentary group clearly professes to take sides with peasant agriculture. Therefore, we clearly refuse the destruction of livelihoods of thousands of family farms through an unfounded tightening of the fertilizer ordinance.”

Members of the status quo coalition also portrayed citizens as victims of stricter regulation, though less frequently and only in the last two periods. Nevertheless, it showed some effort in trying to gain their favor. One occasion is related to the outbreak of the corona pandemic and sudden fears on security of supply. The organization *LSV* wrote in March 2020: “Suspend the drafted fertilizer ordinance. Corona has Germany under control […]. German agriculture is one of the cornerstones of our society. Even if this has become self-evident and has been perceived unconsciously in the past, supplying the population with high quality food is our very own function. We therefore call on the federal government to shift their focus on agriculture’s role for security of supply, so we will still be able to fulfill our function. Everyone should be aware of the systematic relevance.”

The status quo coalition portrayed mostly three groups as villains. The first one comprised EU institutions and this mostly relates to the *EC*. Over time, there was a shift toward German governmental actors on the federal level, including the BMEL, the BMU and the federal government in general. The third group comprised environmental organizations and water associations. Most of these accused villains were part of the reform coalition. However, there were also occasions where actors were being accused as villains who were part of their own coalition or at least had been allies in the past. One of this relates to a press release by the *Freie Bauern* in March 2020 where they expressed their frustration about the adoption of the new fertilizer ordinance and blamed the CDU for their responsibility in this case.

Nevertheless, the results of the congruence networks indicate a high cohesiveness of the status quo coalition in their use of villains and victims. The modularity is around 0 and the density high in all periods (see Table S10). The density varies more strongly between periods. However, this is very likely due to the small number of observations in the first two periods (only 3 and 5 actors, respectively).

To summarize, both coalitions populated their narratives with characters in a way to attract the attention of specific target groups. The status quo coalition mostly appealed to farmers, whereas the reform coalition focused on gaining citizens’ attention. Over time, both coalitions started to compete over the support of small-scale farmers. Finally, both coalitions were very cohesive in their use of villains and victims as a narrative strategy to expand the scope of conflict.

## Discussion

The empirical analysis mostly supports the theoretical expectations on coalition formation and narrative strategies. In this section, the findings are discussed with regard to two strands of literature. The first includes studies on coalition formation in public debates. The second involves literature on policy narratives.

The finding that the coalition in favor of stricter regulation participates more actively in the public debate aligns well with studies on a similar policy issue: the pollution of water by micropollutants. Schaub and Metz (2020) compare coalition formation in discourse and policy networks on a similar German policy subsystem on micropollutants in surface waters. They find that actors with an interest in expanding the scope of conflict are more active in the public debate. Similarly, Schaub and Braunbeck (2020) focus on the German public debate on pharmaceutical residues and find that actors with an interest in containing the scope of conflict, such as the pharmaceutical industry, resort substantially less often to this arena. However, there is also research with different findings. For instance, Leifeld (2013) shows in a study on the German pension reform in 2001 that the public debate in this case was initially dominated by the coalition favoring the policy status quo. An explanation for these differences could be differences in issue salience. Compared to other policy areas, media attention on water protection in Germany has been generally low. In the case of agricultural nitrate pollution, issue salience only increased in the last few years. As Stephan (2020) notes, Schattschneider (1960) already suggested that the dynamics of issue containment and expansion are most likely to occur in policy areas with initially low salience where the start of a widespread debate and increasing public attention would pose a threat to the policy monopoly. Thus, for those who defend the policy status quo, the incentive to avoid the public debate is greater in policy areas with low salience, compared to salient policy areas where public attention is already high. Regarding changes in participation in the public debate, this study finds that the increase in issue salience and likelihood to lose on the issue coincides with increasing participation by the coalition advocating against stricter fertilizer regulation. This not only fits the theoretical expectations, but is also in line with Weible et al. (2020), who generally expect coalitions to grow in size with increasing salience and level of conflict in a policy subsystem. Empirically, the findings on coalition formation are supported by Vogeler et al., (2021a), who apply discourse network analysis to investigate the public debate on agricultural nitrate pollution in a region in northwestern Germany between August 2016 and February 2019. They identify two main coalitions: an agrarian coalition and an environmental coalition, which have very similar actor types compared to those observed at the federal level. Finally, the observed politicization of the German public debate on agricultural nitrate water pollution supports the expectation proposed by Feindt et al. (2020) that in the era of *post-exceptionalism* and increasing priority of environmental protection (Daugbjerg & Feindt, 2017) agricultural politics will become increasingly politicized.

Turning to narrative strategies, the coalitions in this study use both narrative strategies (cost–benefit frames and characters) substantially less frequently to contain the scope of conflict than to expand it. This is not in line with earlier studies on the NPF: McBeth et al. (2007) found that losing coalitions tend to use expanding frames and winning coalitions containing frames in a case study in the US on conflicts over the Yellowstone National Park. Similarly, Shanahan et al. (2013) show in a case study in the US how winning groups in a dispute over wind-farms predominantly use containing frames and the angel shift and losing groups expanding frames and the devil shift. Schlaufer (2018) finds in a Swiss case study that coalitions mobilizing for school reforms tend to use the angel shift whereas those advocating against the reforms resort to the devil shift. However, there are also more recent studies which deviate from this pattern. Merry (2019) finds a predominant use of the angel shift by both winning and losing coalitions in a study on gun control in the US. Other studies do not find associations between winning/losing and containing/expanding strategies (Gottlieb et al., 2018; Heikkila et al., 2014; Stephan, 2020). Nevertheless, many of the studies on the NPF can still identify coalitions based on their narratives. What they have in common is that they can distinguish between coalitions based on their *relative* use of frames and characters. The findings of this study suggest that coalitions differ in how strongly they resort to expanding frames and the devil shift, depending on how likely they are to win or lose on the policy issue.

Similarly to this study, Stephan (2020) observes a predominant use of expanding frames and the devil shift in the Scottish debate on fracking technology. He concludes that efforts of conflict expansion may overshadow those of conflict containment once a political conflict has become *mature* and the debate has gained too much momentum to be closed down. Nevertheless, this does not provide an explanation for the rare use of containing strategies by the status quo coalition at the very beginning of the observation period in this study. Instead, the low frequency of containing narratives at the onset of the conflict might be better explained by the low participation of the status quo coalition at this stage: members of the coalition seem to try to contain the issue by not participating in the debate rather than by disseminating containing policy narratives.

Although the reform coalition changes its behavior over time according to its likelihood to win, its sustained high activity in the debate and continued use of expanding frames at the end of the observation period does not fully align with the theoretical expectations. There might be at least two reasons for this. First, the empirical observation period might not consider the full dynamic developments. One can only speculate, but differences between coalitions might become larger after 2020 in case the stricter regulation will persist. The trends observed in both coalitions’ use of narrative strategies in the last observation period substantiate this thought. Another explanation is borrowed from Stephan (2020) and Pralle (2006): There is indication that both coalitions are not satisfied with the policy status quo in 2020 and both try to expand the scope of conflict to mobilize for policy change, but in different *directions*: the reform coalition advocates for even stricter regulation and the status quo coalition mobilizes for a liberalization of the legal provisions.

Overall, there is evidence provided by this study and some of the previous literature that coalitions use different narrative strategies to try to achieve their policy goals. In this study, this is most apparent by how both coalitions adapt their narrative strategies in response to a changing likelihood to win or lose. Finally, the discourse network analysis of coalitions’ use of villains and victims suggests that members of both coalitions are cohesive in their use of narrative strategies. This is line with Shanahan et al. (2013) who also find high intra-coalitional cohesion in the use of narrative strategies.

## Conclusion

This article set out to investigate the policy conflict over agricultural nitrate pollution of freshwater and stricter fertilizer regulation in Germany between 2010 and 2020. More specifically, it investigated whether actor coalitions differ in their participation in the public debate and their use of narrative strategies in their attempt to influence policy outcomes and whether they adapt their behavior over time in response to changes in the likelihood to win or lose on a policy issue.

The study reveals that the debate on agricultural nitrate pollution of water bodies in Germany became increasingly politicized over the last years. The debate was characterized by an adversarial coalition structure with one actor coalition advocating stricter fertilizer regulation to counter pollution and another mobilizing against the tightening of the regulation. Based on the NPF and early work by Schattschneider (1960) and Baumgartner and Jones (1993), this study suggests that political actors participated strategically in the public debate in their effort to affect policymaking. Both the level of participation and the use of narrative strategies differed between the coalitions. There is strong support that both identified coalitions adapted their narrative strategies over time in response to changes in the likelihood to win or lose on the policy issue resulting from increasing pressure of the EC to adopt stricter fertilizer regulation.

The article provides several important contributions to the study of the NPF. First, it shows that incorporating the study of strategic participation in public debates into the study of policy narratives contributes to a better understanding of differences in narrative strategies between coalitions, especially at the onset of political conflicts. The theoretical argument on strategic participation is based on Schattschneider (1960) and therefore similar to the NPF’s arguments on narrative strategies regarding the scope of conflict. Thus, future studies on the NPF might consider taking up this idea. Furthermore, the article enhances the study of the NPF by more clearly distinguishing between the identification of coalitions and analyzing differences in their narrative strategies. This is achieved through a major methodological contribution. Based on earlier suggestions (Leifeld, 2017; Shanahan et al., 2013; Weible et al., 2016), this study shows that discourse network analysis is a fruitful method to study both coalition formation in a policy subsystem and coalitions’ use of policy narratives. Furthermore, the method not only helps identify coalitions systematically based on congruent policy beliefs, it can also be used to elaborate on the relations between actors and narrative elements, and to investigate how cohesively coalitions construct their policy narratives.

Empirically, the study provides a better understanding of the policy conflict between water protection and the agriculture sector in Germany. It shows how mainly two coalitions, one mobilizing for and the other against stricter fertilizer regulation, try to influence policymaking. Furthermore, it provides a systematic analysis of political actors’ positions on the issue over the course of a decade.

Despite the insights provided, the study has some limitations. First, the findings are based on a single case study. Therefore, they might be case-specific to some extent. Second, narrative strategies are only investigated for non-governmental actors. Thus, the findings on narrative strategies do not apply to governmental actors. A preliminary analysis suggested that governmental actors generally use containing narratives, independent of their membership in one of the two identified coalitions. Thus, comparing governmental and non-governmental actors in their use of narrative strategies by a future study might be insightful. On a similar note, it might be helpful not only to differentiate between different coalitions, but also between different types of coalition members, dependent on their degree of involvement within a coalition as suggested by Weible et al. (2020). This could as well lead to more nuanced findings on political actors’ use of narrative strategies. Third, this study investigates political actors’ strategic behavior as a dependent variable. Thus, the findings do not allow any conclusions of a causal association between this behavior and the policy outcomes in the policy-field. However, future research could build on this study and investigate in a comparative study whether changes in discourse coalitions and in their policy narratives have an impact on policy outcomes.

## Supplementary Information

Below is the link to the electronic supplementary material.

Supplementary file1 (DOCX 1048 kb)

## Footnotes

1 This is indicated by the news coverage of the *Frankfurter Allgemeine Zeitung (FAZ)* on the issue. See also Fig. 4.

2 The CJEU based its ruling on the fertilizer regulation prior to the revision in 2017. However, a procedure based on the revised DüV probably would not have resulted in a different ruling by the CJEU (Härtel, 2018).

3 The following term was used to extract relevant newspaper articles from the *FAZ archive*: “(dünge$, nährstoff$, gülle$, nitrat$, phosphor$) + ($wasser$, $gewässer$, fluss, flüsse, seen, verunreinig$)”.

4 Oftentimes, actors refer to superordinate categories as characters rather than to specific actors. These categories are marked by hashtags in front of the denomination, e.g., *#intensive agriculture*, *#groundwater* or *#biodiversity.*

5 The coalition argues that intensive agriculture threatens farmers’ own means of existence by polluting water and soil and harming ecosystems.

6 Groundwater represents the most important source for drinking water in Germany Umweltbundesamt (2019a).
