# Supplementary material for: Public contestation over agricultural pollution: a discourse network analysis on narrative strategies in the policy process
Source: Policy Sci. 2021 Oct 19;54(4):783–821. doi: 10.1007/s11077-021-09439-x (PMC8523350; doi:10.1007/s11077-021-09439-x)
Supplement: Supplementary file 1 — Supplementary file1 (DOCX 1048 kb) [file 11077_2021_9439_MOESM1_ESM.docx]

# Supplementary materials

## Appendix A: Actor coalitions

Table S1. Full list of actors

| *Actor code* | *Full actor name in German* | *Full actor name in English* | *Type* |
| --- | --- | --- | --- |
| AfD BP | Alternative für Deutschland (Bundespartei) | Alternative for Germany (federal party) | Right-wing party |
| BB BB | Bauernbund Brandenburg | Farmer Federation Brandenburg | Agricultural association |
| BDEW | Bundesverband der Energie- und Wasserwirtschaft | Federal Association of Energy and Water Industry | Water association |
| BMEL | Bundesministerium für Ernährung und Landwirtschaft | Federal Ministry of Food and Agriculture | Federal government |
| BMU | Bundesministerium für Umwelt, Naturschutz und nukleare Sicherheit | Federal Ministry of the Environment, Nature Conservation and Nuclear Safety | Federal government |
| BÖLW | Bundesverband Ökologische Lebensmittelwirtschaft | German Federation of the Organic Food Industry | Organic agriculture |
| BR | Bundesrat | German Federal Council | State government |
| BReg | Bundesregierung | German Federal Government | Federal government |
| BUND | Bund für Umwelt und Naturschutz Deutschland | Friends of the Earth Germany | Environmental organization |
| CDU BP | Christlich Demokratische Union (Bundespartei) | Christian Democratic Union (federal party) | Christian democratic party |
| CSU BP | Christlich-Soziale Union (Bundestagsfraktion) | Christian Social Union in Bavaria (federal parliamentary group) | Christian democratic party |
| DBV | Deutscher Bauernverband | German Farmers' Association | Agricultural association |
| Die Linke BP | Die Linke (Bundespartei) | The Left Federal Party | Left-wing party |
| DNR | Deutscher Naturschutzring | German League for Nature and Environment | Environmental organization |
| DUH | Deutsche Umwelthilfe | Environmental Action Germany | Environmental organization |
| DVGW | Deutscher Verein des Gas- und Wasserfaches | German Association for Gas and Water | Water association |
| DWA | Deutsche Vereinigung für Wasserwirtschaft, Abwasser und Abfall | German Association for Water, Wastewater and Waste | Water association |
| EC | Europäische Kommission | European Commission | European Union |
| FDP BP | Freie Demokratische Partei (Bundespartei) | Liberal Democratic Party (federal party) | Liberal party |
| Greenpeace | Greenpeace | Greenpeace | Environmental organization |
| Grüne BP | Bündnis 90/Die Grünen (Bundespartei) | Alliance '90/The Greens (federal party) | Green party |
| Grüne Liga | Grüne Liga | Green League | Environmental organization |
| HBV | Bauernverband Hessen | Farmers' Association Hessen | Agricultural association |
| HMUKLV | Umwelt- und Agrarministerium Hessen | Ministry of Agriculture and the Environment Hessen | State government |
| IVA | Industrieverband Agrar | German Crop Protection, Pest Control and Fertilizer Association | Agricultural association |
| LSV | Land schafft Verbindung | - | Agricultural association |
| MELUND SH | Landesumweltministerium Schleswig-Holstein | Ministry of the Environment Schleswig-Holstein | State government |
| MLUK BB | Ministerium für Landwirtschaft, Umwelt und Klimaschutz Brandenburg | Ministry of Agriculture, Environment and Climate Protection Brandenburg | State government |
| MULNV NRW | Ministerium für Umwelt, Landwirtschaft, Natur- und Verbraucherschutz des Landes Nordrhein-Westfalen | Ministry of Environment, Agriculture, Nature and Consumer Protection North Rhine-Westphalia | State government |
| NABU | Naturschutzbund | Nature And Biodiversity Conservation Union | Environmental organization |
| NMELV | Ministerium für Ernährung, Landwirtschaft und Verbraucherschutz Niedersachsen | Ministry of Food, Agriculture and Consumer Protection Lower Saxony | State government |
| SPD BP | Sozialdemokratische Partei Deutschlands (Bundespartei) | Social Democratic Party of Germany (federal party) | Social democratic party |
| SRU | Sachverständigenrat für Umweltfragen | German Advisory Council on the Environment | Science |
| UBA | Umweltbundesamt | German Environmental Agency | Federal government |
| VKU | Verband Kommunaler Unternehmen e. V. | Association of Municipal Companies | Water association |
| VZBZ | Verbraucherzentrale (Bundesverband) | Federation of German Consumer Organisations | Consumer protection organization |
| WBAE | Wissenschaftlicher Beirat Agrarpolitik beim Bundesministerium für Ernährung und Landwirtschaft | Agricultural Policy Advisory Council of the Federal Ministry of Food and Agriculture | Science |
| WLV | Westfälisch-Lippischer Landwirtschaftsverband | Agriculture Association Westphalia-Lippe | Agricultural association |
| WWF | World Wide Fund for Nature | World Wide Fund for Nature | Environmental organization |

Table S2. Actor coalitions‘ beliefs indicated by agreement or disagreement for period 1 & 2

|  | | *Period 1* | | | | *Period 2* | | | |
| --- | --- | --- | --- | --- | --- | --- | --- | --- | --- |
|  | | *Reform coalition* | | *Status quo coalition* | | *Reform coalition* | | *Status quo coalition* | |
|  | | *Agree* | *Disagree* | *Agree* | *Disagree* | *Agree* | *Disagree* | *Agree* | *Disagree* |
| *Problem perception* | Conventional farming threatens drinking water quality | 4 |  |  | 1 | 16 |  |  | 3 |
| *Problem perception* | Conventional farming threatens surface water quality | 13 |  |  | 2 | 18 |  |  | 4 |
| *Policy position* | Environmental protection needs organic agriculture | 5 |  |  | 1 | 6 |  |  |  |
| *Policy position* | Tighten Federal Water Act (WHG) |  |  |  |  |  |  |  |  |
| *Policy position* | Tighten Fertilizer Act (DüG) | 6 |  |  | 1 | 13 |  |  | 3 |
| *Policy position* | Tighten Fertilizer Ordinance (DüV) | 10 |  |  | 2 | 18 |  |  | 4 |
| *Policy position* | Tighten Fertilizer Regulation (DüMV) | 2 |  |  |  |  |  |  |  |
| *Policy position* | Tighten regulation on area designation (AVV GeA) |  |  |  |  |  |  |  |  |
| *Policy position* | Tighten regulation on farm gate balance (StoffB) |  |  |  |  |  |  |  |  |
| *Policy instrument* | Limit livestock production to pasture | 2 |  |  |  | 4 |  |  |  |
| *Policy instrument* | Mandatory field-based nutrient accounting |  |  |  |  | 1 |  |  |  |
| *Policy instrument* | Prohibit fertilizer use on ecological compensation areas | 2 |  |  |  |  |  |  |  |
| *Policy instrument* | Stricter blocking periods for fertilizer application | 1 |  |  |  | 8 |  |  | 3 |
| *Policy instrument* | Riparian buffer strips |  |  |  |  | 3 |  |  |  |
| *Policy instrument* | General upper limit on fertilizer application | 1 |  |  | 2 | 8 | 1* |  | 2 |
| *Policy instrument* | Farm gate balance |  |  |  |  | 15 |  |  | 1 |
| *Policy instrument* | Dung exchange („Gülle-Börse“) |  |  |  |  | 1 |  |  |  |
| *Policy instrument* | Environmental tax on nitrate surplus |  |  |  |  | 4 |  |  | 1 |
| *Policy instrument* | Internal differentiation |  |  |  |  |  |  |  |  |

Note: Numbers reproted indicate absolute numbers of actors; * BÖLW deviates from other coalition members.

Table S3. Actor coalitions‘ beliefs indicated by agreement or disagreement for period 3 & 4

|  | | *Period 3* | | | | *Period 4* | | | |
| --- | --- | --- | --- | --- | --- | --- | --- | --- | --- |
|  | | *Reform coalition* | | *Status quo coalition* | | *Reform coalition* | | *Status quo coalition* | |
|  | | *Agree* | *Disagree* | *Agree* | *Disagree* | *Agree* | *Disagree* | *Agree* | *Disagree* |
| *Problem perception* | Conventional farming threatens drinking water quality | 13 | 1* |  | 5 | 14 |  |  | 2 |
| *Problem perception* | Conventional farming threatens surface water quality | 16 |  | 1^●^ | 4 | 16 |  | 1^†^ | 6 |
| *Policy position* | Environmental protection needs organic agriculture | 7 |  |  |  | 8 |  |  | 2 |
| *Policy position* | Tighten Federal Water Act (WHG) |  |  |  |  | 2 |  |  | 2 |
| *Policy position* | Tighten Fertilizer Act (DüG) | 10 |  |  | 1 | 2 |  |  |  |
| *Policy position* | Tighten Fertilizer Ordinance (DüV) | 16 | 1* |  | 9 | 17 |  |  | 8 |
| *Policy position* | Tighten Fertilizer Regulation (DüMV) |  |  |  |  |  |  |  |  |
| *Policy position* | Tighten regulation on area designation (AVV GeA) |  |  |  |  | 5 |  |  | 5 |
| *Policy position* | Tighten regulation on farm gate balance (StoffB) | 10 |  |  | 2 | 8 |  |  | 1 |
| *Policy instrument* | Limit livestock production to pasture | 9 |  | 1^♦^ |  | 11 |  |  |  |
| *Policy instrument* | Mandatory field-based nutrient accounting | 8 |  |  | 5 | 3 |  |  | 2 |
| *Policy instrument* | Prohibit fertilizer use on ecological compensation areas |  |  |  |  |  |  |  |  |
| *Policy instrument* | Stricter blocking periods for fertilizer application | 10 |  |  | 4 | 4 |  |  | 5 |
| *Policy instrument* | Riparian buffer strips | 9 |  |  | 1 | 8 |  |  | 2 |
| *Policy instrument* | General upper limit on fertilizer application | 11 |  |  | 7 | 7 |  |  | 8 |
| *Policy instrument* | Farm gate balance | 11 |  |  | 1 | 7 |  |  |  |
| *Policy instrument* | Dung exchange („Gülle-Börse“) |  |  |  |  |  |  |  |  |
| *Policy instrument* | Environmental tax on nitrate surplus | 1 |  |  |  | 1 |  |  |  |
| *Policy instrument* | Internal differentiation |  |  |  |  | 1^+^ | 6 | 8 |  |

Note: Numbers reproted indicate absolute numbers of actors; *BMEL both agrees and disagrees; +BMEL deviates from other coalition members; †CDU BP deviates from other coalition members; ● NMELV deviates from other coalition members; ♦NMELV deviates from expected coalition position.

## Appendix B: Codebook

Table S4. Cost-benefit frames

|  | Definition | Theory |
| --- | --- | --- |
| Concentrate benefits of stricter regulation | Statement of an actor that implies that a small group benefits from stricter regulation | Actor is against stricter regulation  and losing |
| Diffuse costs of  stricter regulation | Statement of an actor that implies that a large group is harmed by stricter regulation | Actor is against stricter regulation  and losing |
| Diffuse benefits of liberalization | Statement of an actor that implies that a large group benefits from liberalization | Actor is in favor of liberalization  and winning |
| Concentrate costs of liberalization | Statement of an actor that implies that a small group is harmed by liberalization | Actor is in favor of liberalization  and winning |
| Concentrate benefits of liberalization | Statement of an actor that implies that a small group benefits from liberalization | Actor is against liberalization  and losing |
| Diffuse costs of liberalization | Statement of an actor that implies that a large group is harmed by liberalization | Actor is against liberalization  and losing |
| Diffuse benefits of stricter regulation | Statement of an actor that implies that a large group benefits from stricter regulation | Actor is in favor of stricter regulation and winning |
| Concentrate costs of stricter regulation | Statement of an actor that implies that a small group is harmed by stricter regulation | Actor is in favor of stricter regulation and winning |

Note: Based on Shanahan et al. (2013) and Shanahan et al. (2018).

Table S5. Characters

|  | Definition | Theory |
| --- | --- | --- |
| Hero | The entity designated as fixing or being able to fix the specified problem. Those who take action with purpose to achieve or oppose a policy solution | Actor is winning |
| Villain | Those who create a harm, or inflicts damage or pain upon a victim or, in other cases as one who opposes the aims of the hero. | Actor is losing |
| Victim | Those who are harmed by a particular action or inaction. | Actor is losing |
| Beneficiary | Those who profit from a particular action or inaction. | Actor is winning |

Note: Based on Shanahan et al. (2013), Shanahan et al. (2018) and Weible et al. (2016).

*References*

Shanahan, E. A., Jones, M. D., McBeth, M. K., & Lane, R. R. (2013). An Angel on the Wind: How Heroic Policy Narratives Shape Policy Realities. *Policy Studies Journal*, *41*(3), 453–483.

Shanahan, E. A., Jones, M. D., & McBeth, M. K. (2018). How to conduct a Narrative Policy Framework study. *The Social Science Journal*, *55*(3), 332–345.

Weible, C. M., Olofsson, K. L., Costie, D. P., Katz, J. M., & Heikkila, T. (2016). Enhancing Precision and Clarity in the Study of Policy Narratives: An Analysis of Climate and Air Issues in Delhi, India. *Review of Policy Research*, *33*(4), 420–441.

## Appendix C: Cost-benefit-frames

Table S6. Results of parametric and non-parametric tests for coalition differences in mean contain-expand ratio

|  |  | *Period 1* |  | *Period 2* |  | *Period 3* |  | *Period 4* |  |
| --- | --- | --- | --- | --- | --- | --- | --- | --- | --- |
| *Status quo coalition* | *observations* | 5 |  | 20 |  | 40 |  | 98 |  |
|  | *mean* | 0.600 |  | 0.050 |  | -0.184 |  | -0.452 |  |
| *Reform coalition* | *observations* | 14 |  | 88 |  | 150 |  | 92 |  |
|  | *mean* | -0.643 |  | -0.568 |  | -0.567 |  | -0.337 |  |
|  | *difference in means* | 1.243 | *** | 0.618 | *** | 0.383 | *** | -0.115 |  |
|  | *test* | t-test |  | Welch t-test |  | t-test |  | t-test |  |
|  | *t-value* | 4.682 |  | 3.207 |  | 3.652 |  | -1.386 |  |
|  | *p-value* | 0.000 |  | 0.004 |  | 0.000 |  | 0.167 |  |
|  | *test* | Wilcoxon rank sum |  | Wilcoxon rank sum |  | Wilcoxon rank sum |  | Wilcoxon rank sum |  |
|  | *W* | 65 |  | 1248.5 |  | 3432.5 |  | 4154 |  |
|  | *p-value* | 0.003 |  | 0.001 |  | 0.003 |  | 0.235 |  |
| Note: stars indicate level of statistical significance: *** p < 0.01, ** p < 0.05, * p < 0.1; Welch t-tests have been computed instead of t-tests in cases where the assumption of variance homogeinity was violated. | | | | | | | | | |

Table S7. Results of Mann-Kendall Trend Tests for mean frame ratio per month

|  |  | *Period 1 & 2* |  | *Period 3 & 4* |  |
| --- | --- | --- | --- | --- | --- |
| *Status quo coalition* | *Observations (months)* | *87* |  | *45* |  |
|  | *Kendall's tau* | 0.049 |  | -0.436 |  |
|  | *Score* | 102 |  | -370 |  |
|  | *p-value* | 0.572 |  | 0.000 | *** |
| *Reform coalition* | *Observations (months)* | *87* |  | *45* |  |
|  | *Kendall's tau* | -0.354 |  | 0.201 |  |
|  | *Score* | -921 |  | 190 |  |
|  | *p-value* | 0.000 | *** | 0.062 | * |
| Note: *** p < 0.01, ** p < 0.05, * p < 0.1. | |  |  |  |  |


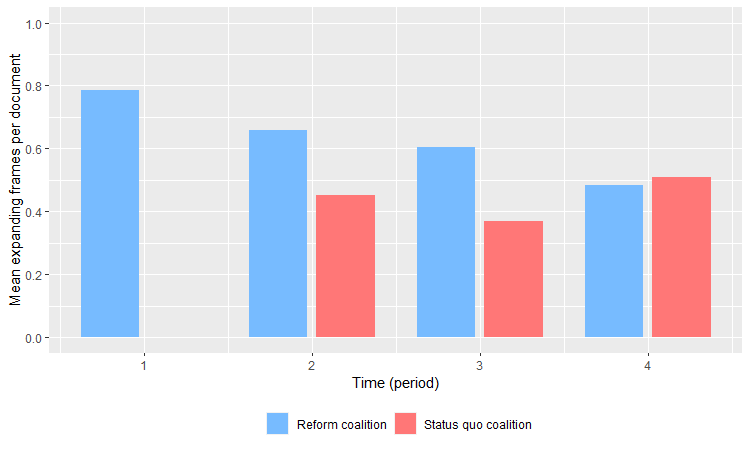


Figure S1. Mean number of expanding frames within documents per period, plotted separately for each coalition.


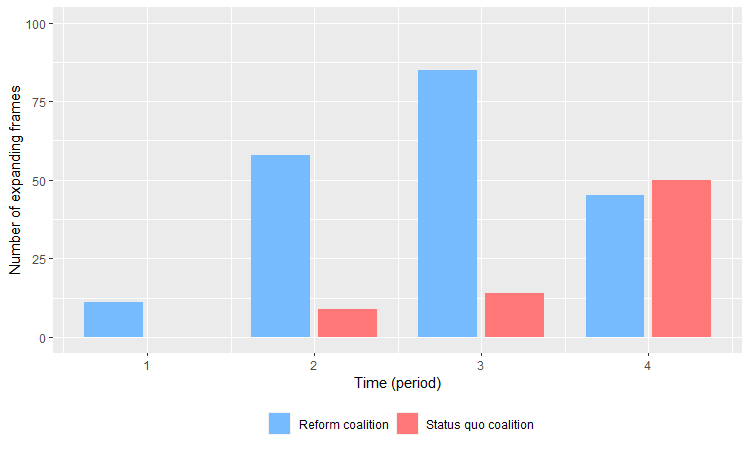


Figure S2. Absolute number of expanding frames per period, plotted separately for each coalition.


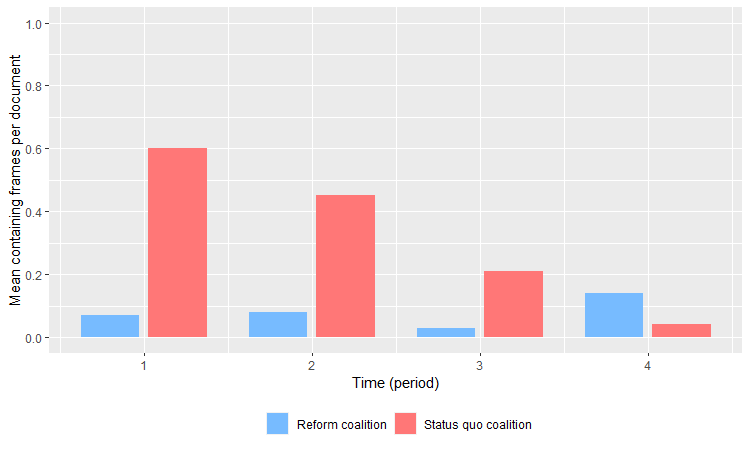


Figure S3. Mean number of containing frames within documents per period, plotted separately for each coalition.


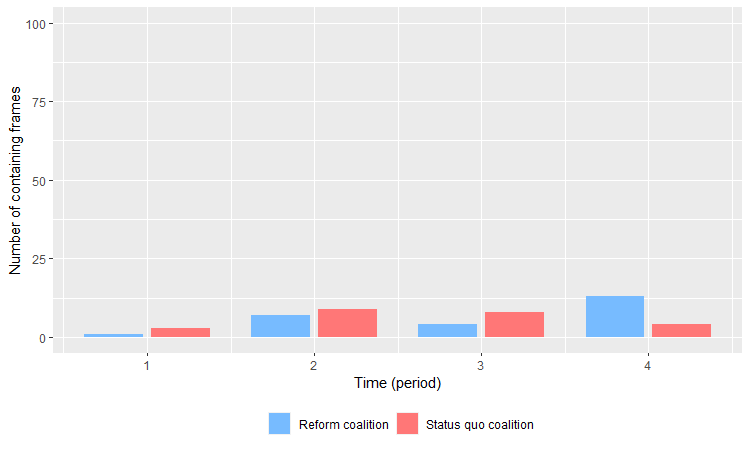


Figure S4. Absolute number of containing frames per period, plotted separately for each coalition.


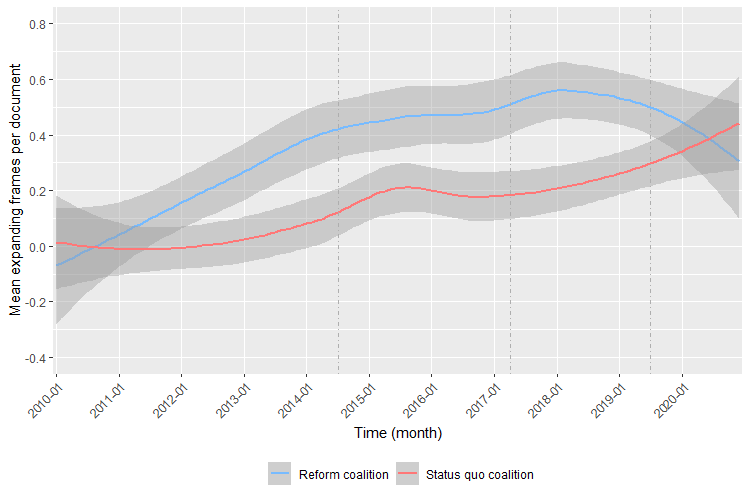


Figure S5. Changes in coalition’s use of expanding frames over time.

Note: The graph reports smoothing lines estimated by using the non-parametric LOESS (locally estimated scatterplot smoothing) method based on mean number of expanding frames within documents per month. The grey shaded areas around the lines represent 90% confidence intervals.

## Appendix D: Devil shift-angel shift

Table S8. Results of parametric and non-parametric tests for coalition differences in mean hero-villain ratio

|  |  | *Period 1* |  | *Period 2* |  | *Period 3* |  | *Period 4* |  |
| --- | --- | --- | --- | --- | --- | --- | --- | --- | --- |
| *Status quo coalition* | *observations* | 5 |  | 20 |  | 40 |  | 98 |  |
|  | *mean* | -0.667 |  | -0.183 |  | -0.165 |  | -0.495 |  |
| *Reform coalition* | *observations* | 14 |  | 88 |  | 150 |  | 92 |  |
|  | *mean* | -0.345 |  | -0.633 |  | -0.666 |  | -0.457 |  |
|  | *difference in means* | -0.321 |  | 0.449 | *** | 0.501 | *** | -0.038 |  |
|  | *test* | t-test |  | t-test |  | Welch t-test |  | t-test |  |
|  | *t-value* | -0.924 |  | 3.007 |  | 3.983 |  | -0.480 |  |
|  | *p-value* | 0.369 |  | 0.003 |  | 0.000 |  | 0.632 |  |
|  | *test* | Wilcoxon rank sum |  | Wilcoxon rank sum |  | Wilcoxon rank sum |  | Wilcoxon rank sum |  |
|  | *W* | 26.5 |  | 1182 |  | 4166.5 |  | 4274 |  |
|  | *p-value* | 0.429 |  | 0.006 |  | 0.000 |  | 0.483 |  |
| Note: stars indicate level of statistical significance: *** p < 0.01, ** p < 0.05, * p < 0.1. Welch t-tests have been computed instead of t-tests in cases where the assumption of variance homogeinity was violated. | | | | | | | | | |

Table S9. Results of Mann-Kendall Trend Tests for mean hero-villain ratio per month per coalition

|  |  | *Period 1 & 2* |  | *Period 3 & 4* |  |
| --- | --- | --- | --- | --- | --- |
| *Status quo coalition* | *Observations (months)* | *87* |  | *45* |  |
|  | *Kendall's tau* | -0.196 | ** | -0.423 | *** |
|  | *Score* | -405 |  | -363 |  |
|  | *p-value* | 0.024 |  | 0.000 |  |
| *Reform coalition* | *Observations (months)* | *87* |  | *45* |  |
|  | *Kendall's tau* | -0.358 | *** | 0.405 | *** |
|  | *Score* | -983 |  | 385 |  |
|  | *p-value* | 0.000 |  | 0.000 |  |
| Note: stars indicate level of statistical significance: *** p < 0.01, ** p < 0.05, * p < 0.1 | | | | | |

Table S10. Actor coalitions‘ cohesiveness in their use of villains and victims

|  |  | *Period 1* | *Period 2* | *Period 3* | Period 4 |
| --- | --- | --- | --- | --- | --- |
| *Status quo coalition* | *observations* | 3 | 5 | 8 | 9 |
|  | *density* | 0.333 | 1 | 0.714 | 0.778 |
|  | *modularity* | 0 | 0 | 0 | 0.009 |
| *Reform coalition* | *observations* | 9 | 12 | 13 | 10 |
|  | *density* | 0.444 | 0.833 | 0.833 | 0.644 |
|  | *modularity* | 0 | 0.034 | 0.013 | 0 |

Note: One-mode congruence networks with statements filtered at document level, normalized by average activity.

Table S11. Actor coalitions‘ cohesiveness in their use of all characters

|  |  | *Period 1* | *Period 2* | *Period 3* | Period 4 |
| --- | --- | --- | --- | --- | --- |
| *Status quo coalition* | *observations* | 3 | 5 | 8 | 9 |
|  | *density* | 0.333 | 1 | 0.5 | 0.75 |
|  | *modularity* | 0 | 0 | 0 | 0.006 |
| *Reform coalition* | *observations* | 9 | 12 | 13 | 10 |
|  | *density* | 0.278 | 0.742 | 0.769 | 0.400 |
|  | *modularity* | 0.071 | 0.024 | 0.004 | 0 |

Note: One-mode congruence networks with statements filtered at document level, normalized by average activity.


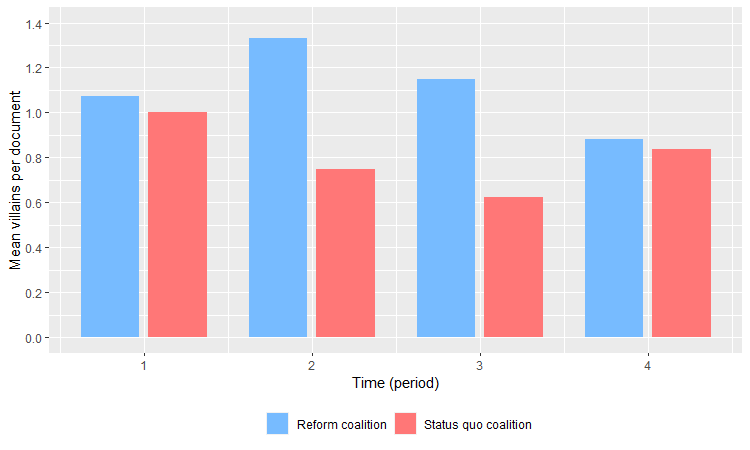


Figure S6. Mean number of villains within documents per period, plotted separately for each coalition


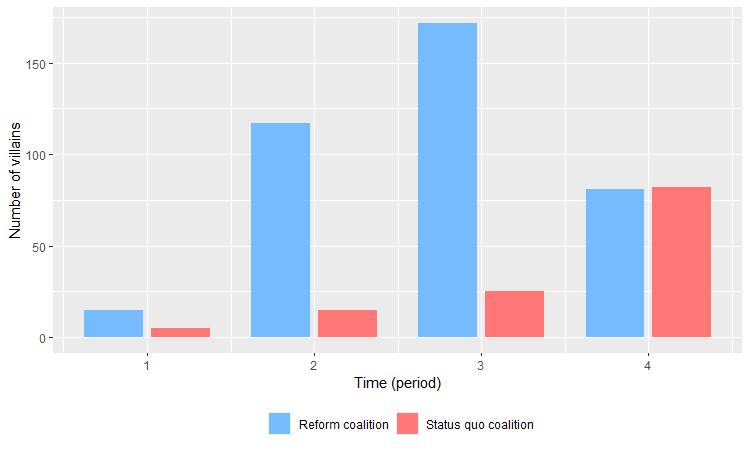


Figure S7. Absolute number of villains per period, plotted separately for each coalition


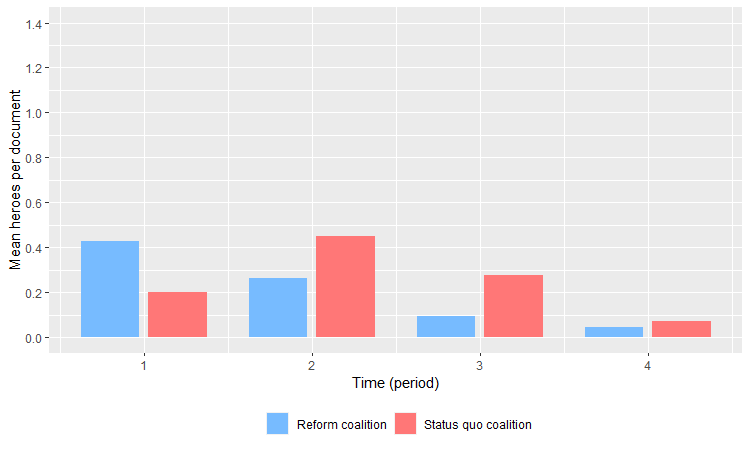


Figure S8. Mean number of heroes within documents per period, plotted separately for each coalition


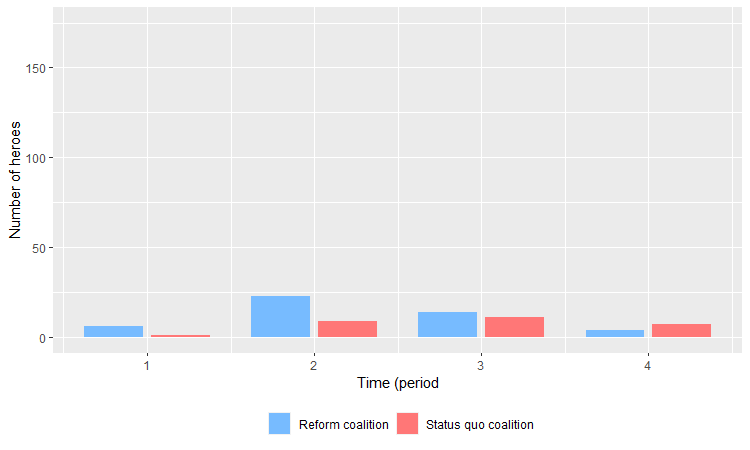


Figure S9. Number of heroes per period, plotted separately for each coalition


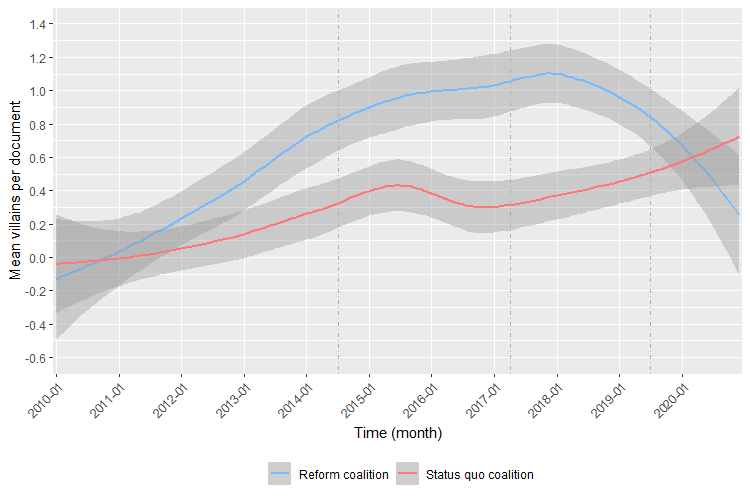


Figure S10. Changes in coalition’s use of villains over time.

Note: The graph reports smoothing lines estimated by using the non-parametric LOESS (locally estimated scatterplot smoothing) method based on mean villains within documents per month. The grey shaded areas around the lines represent 90% confidence intervals.


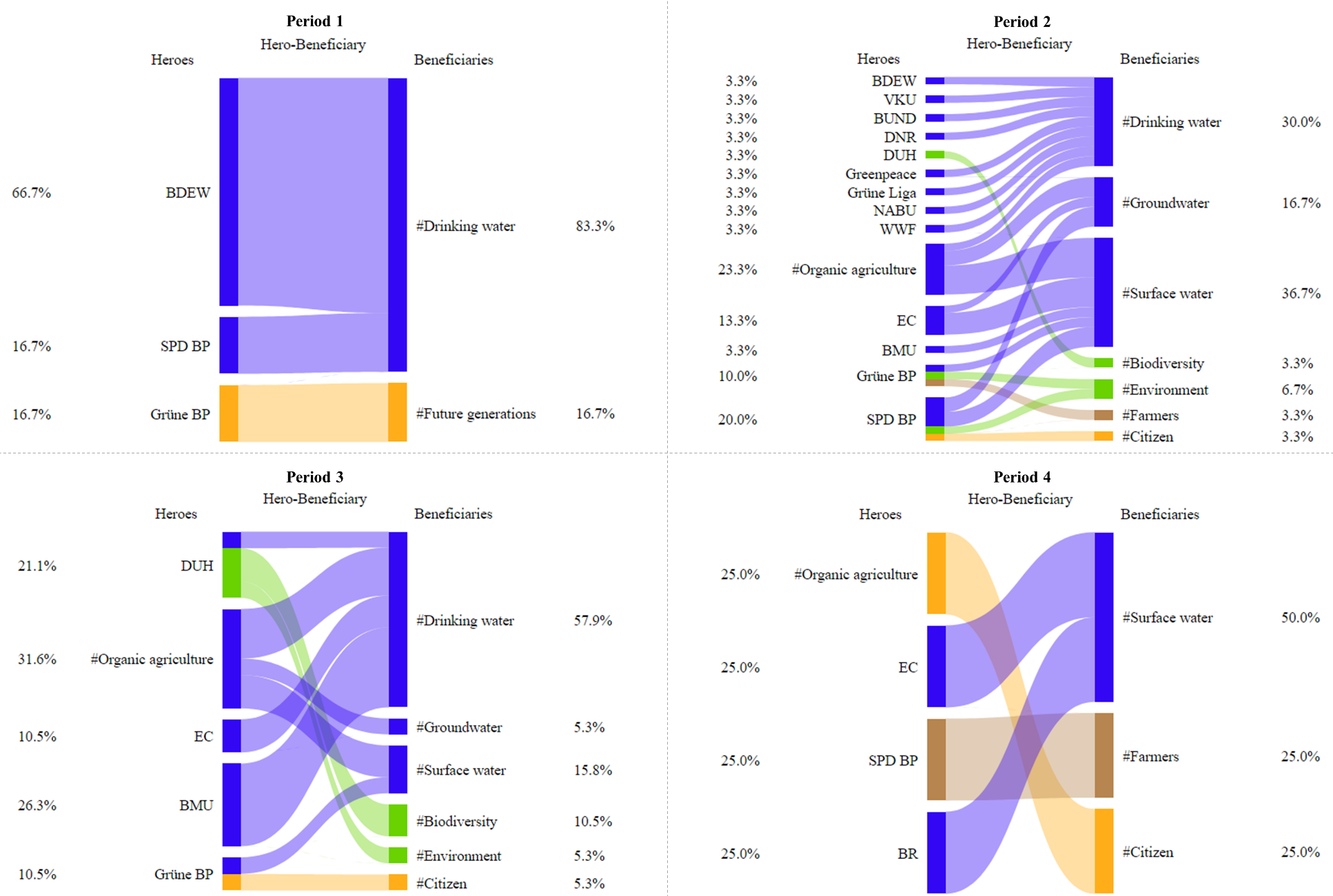


Figure S11. Use of heroes and beneficiaries by reform coalition


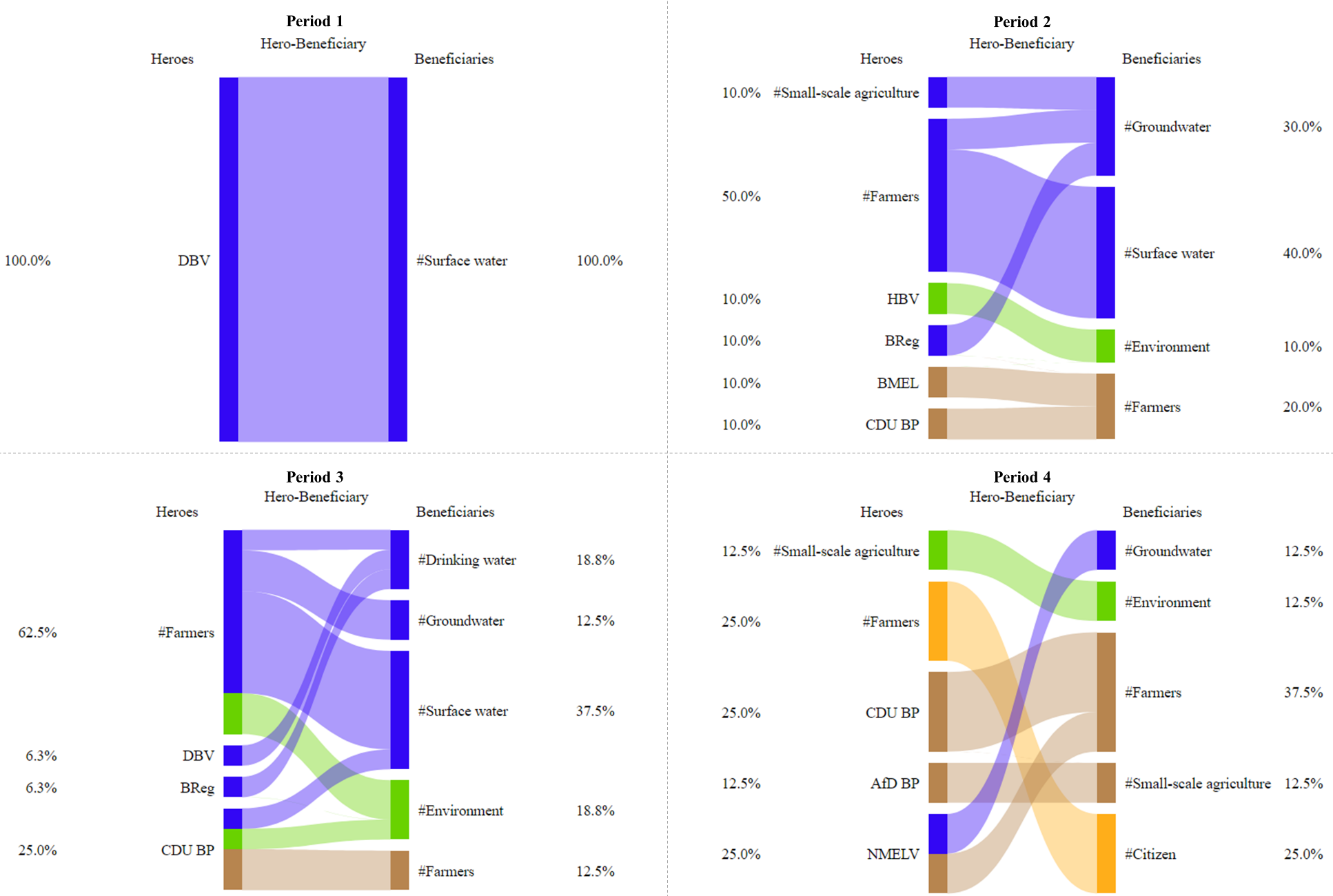


Figure S12. Use of heroes and beneficiaries by status quo coalition
